# Anticoagulant mechanism, pharmacological activity, and assessment of preclinical safety of a novel fibrin(ogen)olytic serine protease from leaves of *Leucas indica*

**DOI:** 10.1038/s41598-018-24422-y

**Published:** 2018-04-18

**Authors:** Debananda Gogoi, Neha Arora, Bhargab Kalita, Rahul Sarma, Taufikul Islam, Sidhhartha S. Ghosh, Rajlakshmi Devi, Ashis K. Mukherjee

**Affiliations:** 1 Microbial Biotechnology and Protein Research Laboratory, Department of Molecular Biology and Biotechnology, School of Sciences, Tezpur University, Tezpur, 784028 Assam India; 2 Department of Biosciences and Bioengineering, Indian Institute of Technology, Guwahati, 781039 Assam India; 3 Biochemistry Laboratory, Life Sciences Division, Institute of Advanced Study in Science and Technology, Guwahati, 781035 Assam India

## Abstract

The harnessing of medicinal plants containing a plethora of bioactive molecules may lead to the discovery of novel, potent and safe therapeutic agents to treat thrombosis-associated cardiovascular diseases. A 35 kDa (m/z 34747.5230) serine protease (lunathrombase) showing fibrin(ogen)olytic activity and devoid of N- and O- linked oligosaccharides was purified from an extract of aqueous leaves from *L. indica*. The LC-MS/MS analysis, *de novo* sequencing, secondary structure, and amino acid composition determination suggested the enzyme’s novel characteristic. Lunathrombase is an αβ-fibrinogenase, demonstrating anticoagulant activity with its dual inhibition of thrombin and FXa by a non-enzymatic mechanism. Spectrofluorometric and isothermal calorimetric analyses revealed the binding of lunathrombase to fibrinogen, thrombin, and/or FXa with the generation of endothermic heat. It inhibited collagen/ADP/arachidonic acid-induced mammalian platelet aggregation, and demonstrated antiplatelet activity via COX-1 inhibition and the upregulation of the cAMP level. Lunathrombase showed *in vitro* thrombolytic activity and was not inhibited by endogenous protease inhibitors α_2_ macroglobulin and antiplasmin. Lunathrombase was non-cytotoxic to mammalian cells, non-hemolytic, and demonstrated dose-dependent (0.125–0.5 mg/kg) *in vivo* anticoagulant and plasma defibrinogenation activities in a rodent model. Lunathrombase (10 mg/kg) did not show toxicity or adverse pharmacological effects in treated animals.

## Introduction

Cardiovascular diseases (CVDs) such as myocardial infarction, stroke, deep-vein thrombosis, and pulmonary embolism are major causes of mortality worldwide^1,2^. The haemostatic system requires a balance between fibrin formation (coagulation) and fibrin dissolution (fibrinolysis) to prevent the free flow of blood at sites of injury and to ensure the perfusion of blood through tissues^3^. Factor Xa and thrombin are recognized as indispensable components of the coagulation cascade^4^. FXa is the major component of the prothrombinase complex, comprised of factor Va, negatively charged phospholipids, and calcium ions^5^. The prothrombinase complex eventually converts inactive prothrombin to active thrombin for the conversion of soluble fibrinogen into insoluble fibrin polymer (clot), which is ultimately degraded by plasmin^4,6^. Any disruption in this delicate balance leads to thrombosis and/or hemorrhage that results in disseminated intravascular coagulopathy (DIC), which poses a clinical challenge for treatment.

Higher levels of fibrinogen (hyperfibrinogenemia) have been reported to alter the hemodynamic properties of blood that subsequently enhance the intravascular fibrin deposition and pose as an independent risk factor for both arterial and venous thrombosis^7,8^. Higher levels of fibrinogen have also been reported to induce lipid proliferation that initiates the development of atherosclerosis, resulting in ischemic pathology^9^. Therefore, anticoagulant fibrinogenolytic enzymes capable of inhibiting thrombin have proven to be effective in preventing thrombosis^10–14^ and treating hyperfibrinogenemia-associated disorders^15,16^. Such anticoagulant molecules need to be cost-effective and preferably devoid of the risk of hemorrhage, allergic reactions, and other adverse pharmacological complications seen in most of the commercial anticoagulant cardiovascular drugs^17,18^.

Herbs containing antithrombotic activities have been suggested to act as medicinal plants that could lead to the discovery of novel therapeutic agents for treating thrombosis-associated diseases^19–23^. The plant *Leucas indica*, belonging to the Lamiaceae family is mostly used in folk medicine for treating asthma and as decoctions in traditional medicine to reduce nasal congestion. Interestingly, studies from our laboratory have discovered the presence of anticoagulant fibrinogenolytic enzyme(s) in the aqueous leaf extracts of this plant. To the best of our knowledge, this is the first report on the biochemical and pharmacological characterization, and elucidation of the anticoagulant mechanism of a fibrin(ogen)olytic serine protease purified from the aqueous leaf extract of *L. indica*. This plant-derived fibrinogenolytic serine protease demonstrated dual inhibition of thrombin and FXa, and did not show *in vivo* toxicity in experimental animals which has never before been demonstrated for any protease, and the finding suggests its therapeutic application as an anticoagulant, antithrombotic drug.

## Results

### Lunathrombase is a major fibrinogenolytic protease purified from the leaves of *L. indica*

**Figure 1:**
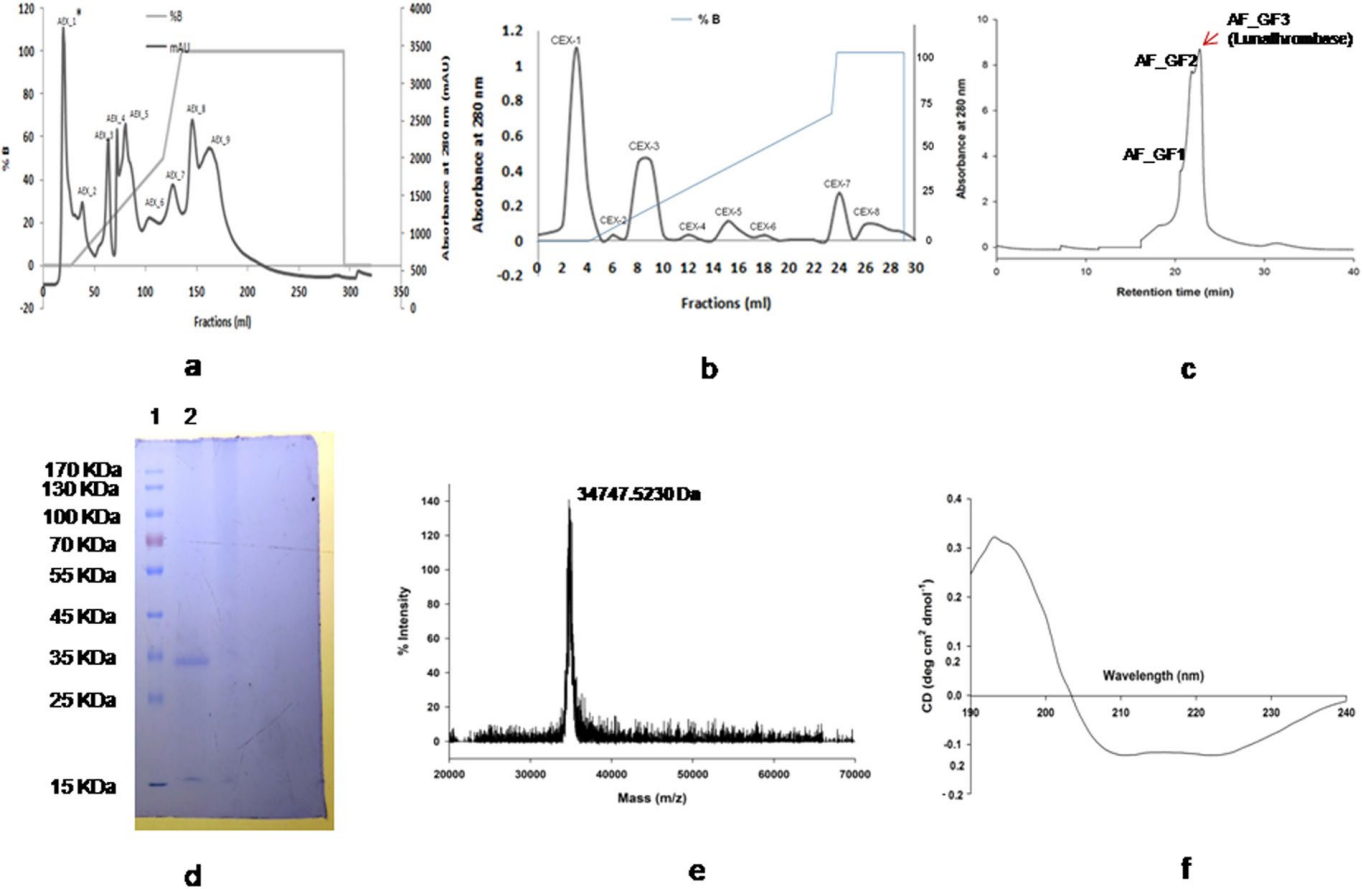
(**a**) Fractionation of crude aqueous shade leave extract of *L. indica* on a PrepTM anion exchange DEAE-cellulose FF 16/10 column. After washing the column with two volume of equilibration buffer (20 mM K.P buffer, pH 7.4),the bound fraction were eluted with a linear gradient of 0.1–1.0 M NaCl in 20 mM K.P buffer at pH 7.4 at a flow rate of 1.0 ml/min. The elution profile was monitored at 280 nm. The first peak (AEX_1) corresponds to the elution of fraction showing highest anticoagulant and fibrin(ogeno)lytic activities. (**b**) Fractionation of the anion-exchange unbound fraction (AEX_1 peak) on cation exchange CM-cellulose (20 mm × 60 mm) column. After washing the column with two volume of equilibration buffer (20 mM K.P buffer, pH 7.4), the bound fraction were eluted with a linear gradient of 0.1–1.0 M NaCl in 20 mM K.P buffer at pH 7.4 at a flow rate of 0.5 ml/min. The elution profile was monitored at 280 nm. The *L. indica* first peak (CEX_1) corresponds to the elution of fraction showing highest anticoagulant and fibrin(ogeno)lytic activities. (**c)** Gel filtration of the CEX_1 on Shodex KW-803 column (5 µm, 8 × 300 mm). After washing the column with two volume of equilibration buffer (20 mM K.P buffer, containing 150 mM NaCl pH 7.4). Elution was carried out with equilibration buffer at a flow rate of 0.5 ml/min. The red arrow indicates elution of lunathrombase (**d**) Determination of purity and molecular mass of AF_GF3 (lunathrombase) by 12.5% SDS-PAGE; Lane 1, protein molecular markers; lane 2, reduced lunathrombase (20.0 µg). (**e)** MALDI-ToF mass spectra of lunathrombase (5.0 µg). (**f**) Circular dichroism (CD) spectra of lunathrombase. Native lunathrombase (0.3 mg/ml) was dissolved in 20 mM potassium phosphate buffer pH 7.0 and the far UV-CD spectra was recorded at room temperature (~25 °C) between 190 and 240 nm against the appropriate buffer (blank). The original unedited gel of Fig. 1d is shown in Supplementary Figure 22.

Fractionation of crude aqueous leaf extracts of *L. indica* through an anion exchange matrix resulted in separation of proteins into nine peaks (Fig. 1a). Peak1 (AEX_1) eluted with the equilibration buffer (unbound fractions) and showed significant fibrinogenolytic and anticoagulant activities. Cation-exchange chromatography was used for the AEX_1 fraction, which was separated into eight fractions (CEX_1 to CEX_8) (Fig. 1b). The unbound peak CEX_1 eluted with the equilibration buffer demonstrated significant fibrinogenolytic and anticoagulant activities. HPLC gel filtration of CEX_1 fraction resolved it in three protein peaks (AF_GF1 to AF_GF3); the AF_GF3 fractions eluted in tube no. 45 to 48 with retention time 23 to 24 min showed highest fibrinogenolytic activity (Fig. 1c). The SDS-PAGE (reduced) analysis of 20 µg of protein from the AF_GF3 peak proteins revealed a single, distinct band for a 35 kDa protein (Fig. 1d), which was named lunathrombase. By MALDI-ToF-MS analysis lunathrombase showed a single sharp peak at m/z 34767.52 Da indicating purity of preparation (Fig. 1e). The summary of purification of lunathrombase is shown in Supplementary Table S1. The anticoagulant and fibrinogenolytic activity of all the gel filtration fractions were found to be lower as compared to CEX_1 fraction which was due to other low molecular mass phytochemicals present in this fraction (CEX_1) that contributed to anticoagulant activity. Further, the combined fibrinogenolytic activity of all the three gel filtration fractions results in higher specific activity of cation exchange fraction CEX_1.

### Peptide mass fingerprinting, *de novo* sequencing, amino acid composition, and secondary structure analyses of the unique lunathrombase

Tandem mass spectroscopic analysis and *de novo* sequencing of lunathrombase did not reveal its similarity with any plant protein, suggesting that it may be a new plant protein. Nevertheless, one of the MS-MS-derived tryptic fragments of lunathrombase (IITHPNFNGNTLDNDIMLIK) demonstrated a conserved domain belonging to the trypsin-like superfamily suggesting that lunathrombase may be a previously uncharacterized plant protease. The alignment of IITHPNFNGNTLDNDIMLIK with other trypsin (-like) enzymes is shown in Supplementary Fig. S1.

Analysis of the amino acid composition of lunathrombase using Swiss-prot and TrEMBL databases did not reveal any similarity with other proteins from plants (Supplementary Table S2). The combined results indicate that lunathrombase is a novel protease from *L*. *indica*. In addition, analysis of the CD spectrum of lunathrombase demonstrated that it consists of 23.4% α-helix, 17% beta sheets, with a turn of 28.2% and 31% random coils (Fig. 1f).

### Lunathrombase demonstrated anticoagulant and fibrin(ogen)olytic activity and did not contain N- or O-linked oligosachharides

**Figure 2:**
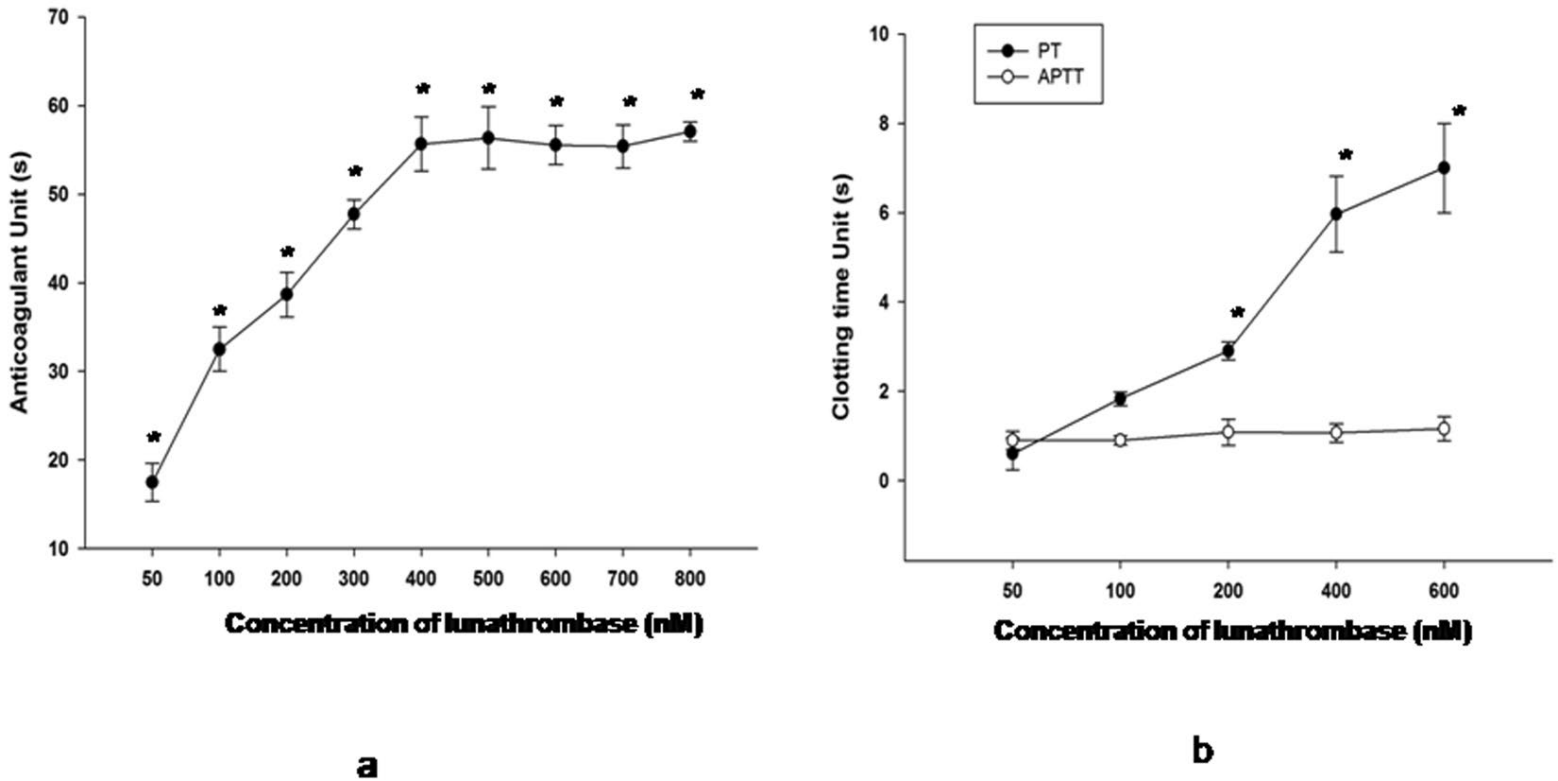
(**a**) Dose- dependent *in vitro* anticoagulant activity of lunathrombase against human platelet-poor plasma. (**b**) Effect of lunathrombase (50–600 nM) on APTT and PT of PPP isolated from human blood. Values are mean ± S.D. of triplicate determinations. Significance of difference with respect to control (without lunathrombase) *p < 0.05.

Lunathrombase dose-dependently prolonged the Ca^2+^ clotting time of PPP, and at a concentration of 400 nM, saturation in anticoagulant activity was observed (Fig. 2a). Lunathrombase demonstrated optimum anticoagulant activity at 10 min of pre-incubation with PPP (Supplementary Fig. S2). At a concentration of 500 nM, lunathrombase did not affect APTT, though it significantly (p < 0.05) enhanced the PT of PPP (Fig. 2b).

**Figure 3:**
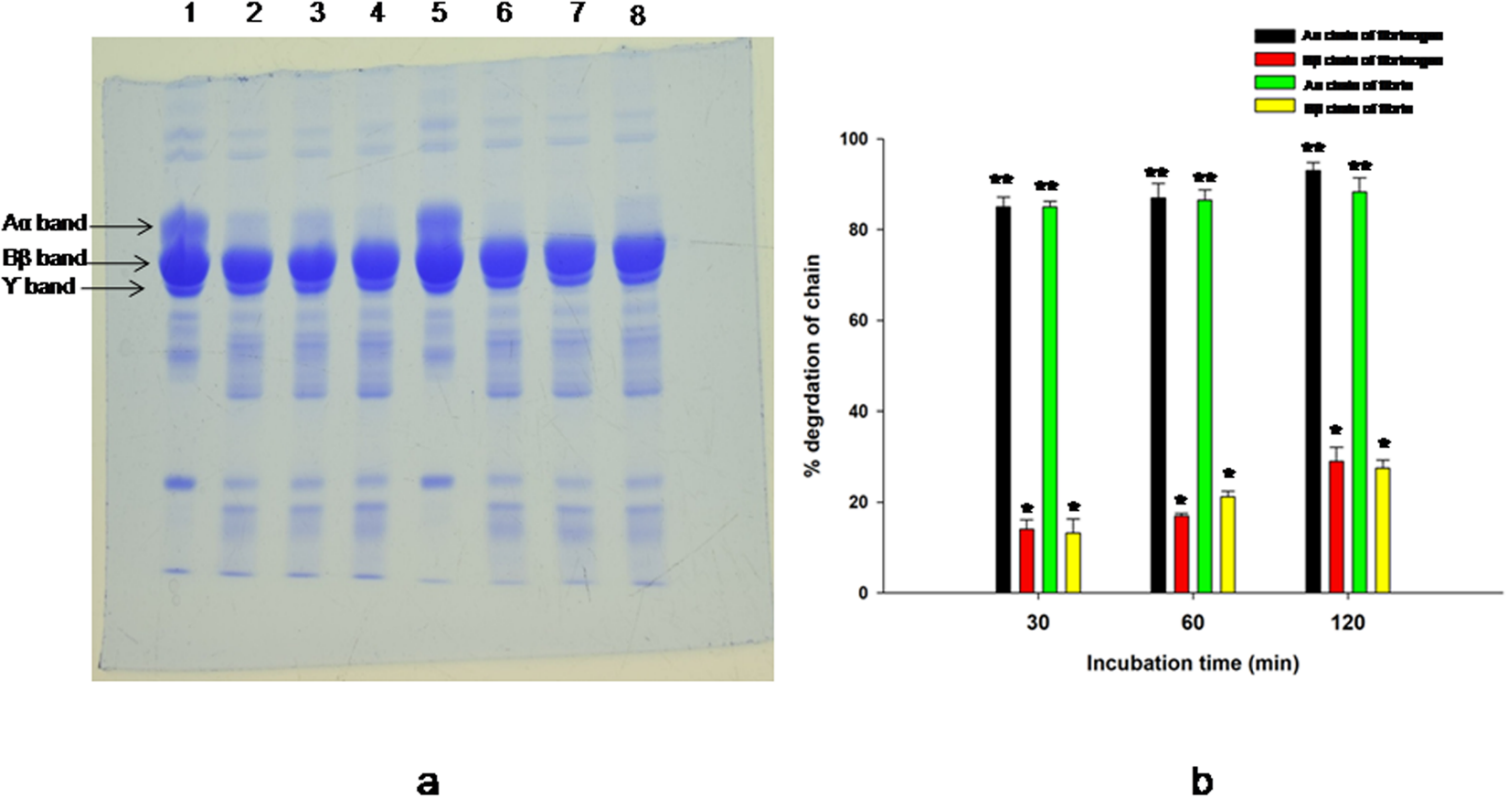
(**a**) Kinetics of fibrinogenolytic activity of lunathrombase. The degradation products were separated by 12.5% SDS-PAGE (reducing conditions). Lane 1, control human fibrinogen (0.25% w/v in 20 mM K-phosphate buffer, 150 mM NaCl, pH 7.4); lanes 2–4, human fibrinogen treated with lunathrombase (0.2 µM) for 30, 60 and 120 min, respectively, at 37 °C, pH 7.4. Kinetics of fibrinolytic activity of lunathrombase. Lane 5, control human fibrin; lanes 6–8, human fibrin treated with 0.2 µM lunathrombase for 30, 60 and 120 min, respectively, at 37 °C, pH 7.4. (**b**) Densitometry analysis to determine the percent degradation of Aα- and Bβ- chains of fibrinogen/fibrin. Significance of difference with respect to control Aα chain of fibrinogen/fibrin (0% degradation), **p < 0.01; Significance of difference with respect to control Bβ chain of fibrinogen/fibrin (0% degradation) *p < 0.05.

Lunathrombase showed dose- and time-dependent fibrin(ogen)olytic activity. The kinetics of fibrinogen/fibrin degradation indicated that lunathrombase preferentially degraded the Aα chain of fibrinogen/fibrin (Fig. 3a,b). With an increase in incubation time to 120 min, the Bβ chain was slowly removed; however, the γ-chain of fibrinogen/fibrin remained intact after 2 h of incubation at 37 °C (Fig. 3a,b). The fibrinogenolytic activity of lunathrombase was found to be superior to Nattokinase, plasmin, and thrombin (Supplementary Fig. S3); whereas, the fibrinolytic activity of lunathrombase surpassed that of Nattokinase, streptokinase, and plasmin under identical experimental conditions (Supplementary Fig. S4). The *Km and Vmax* values of lunathrombase towards fibrinogen were determined to be 52.64 ± 9.8 µM and 52.33 ± 5.7 µM/min (mean ± SD), respectively (Supplementary Fig. S4) whereas the *Km and Vmax* values of Nattokinase towards fibrinogen were determined at 2.88 ± 1.1 µM and 1.97 ± 0.5 µM/min, respectively (Supplementary Fig. S6). The RP-HPLC analysis indicated that lunathrombase and Nattokinase perhaps cleaves different sites of fibrinogen resulting in different elution profiles of the fibrinogen/fibrin degradation products from the RP-HPLC column (Supplementary Fig. S7).

The lunathrombase-mediated degradation of fibrinogen in the presence of FPA or FPB, or with both FPA and FPB, did not result in the inhibition of fibrin(ogeno)lytic activity, when compared to controls (without fibrinopeptides) (data not shown). This result suggests that free FPA or FPB do not influence the fibrin(ogeno)lytic activity of lunathrombase. Lunathrombase did not hydrolyze albumin or globulin (data not shown). It showed optimum fibrin(ogen)olytic activity at 35–37 °C and at pH 7.0–7.4 (Supplementary Figs S8 and S9). Lunathrombase demonstrated BAEE and TAME hydrolyzing activity with specific activities of 436 ± 9.5 and 321 ± 11.2 U/mg (mean ± SD, n = 3), respectively. Lunathrombase contained 7.0% of neutral sugar, though it did not contain N- or O-linked oligosaccharides (Supplementary Fig. S10).

### Inhibitor study shows lunathrombase is a serine protease and its activity is not influenced by the endogenous protease inhibitors of plasma

The fibrin(ogen)olytic activity of lunathrombase was not affected (p > 0.05) by any of the tested metal ions (Supplementary Fig. S11), though it was inhibited by PMSF (a serine protease inhibitor), iodoacetamide (a cysteine protease inhibitor), and pBPB (a histidine inhibitor) (Supplementary Table S3 and Supplementary Fig. S12). Nevertheless, EDTA (a metalloprotease inhibitor) and DTT (a disulfide bond reducing agent) failed to inhibit the fibrin(ogen)olytic activity of lunathrombase (Supplementary Table S3, Supplementary Fig. S12). The SDS-PAGE analysis suggested that lunathrombase at the tested dose was unable to degrade extracellular matrix proteins, namely Type-IV collagen, laminin, and fibronectin at physiological conditions (37 °C, pH 7.4) (Supplementary Fig. S13). Further, the endogenous protease inhibitors, α_2_ macroglobulin and antiplasmin, did not inhibit the fibrin(ogen)olytic activity of lunathrombase (data not shown).

### Lunathrombase inhibits the pharmacological activity of the blood coagulation factors, thrombin and FXa

**Figure 4:**
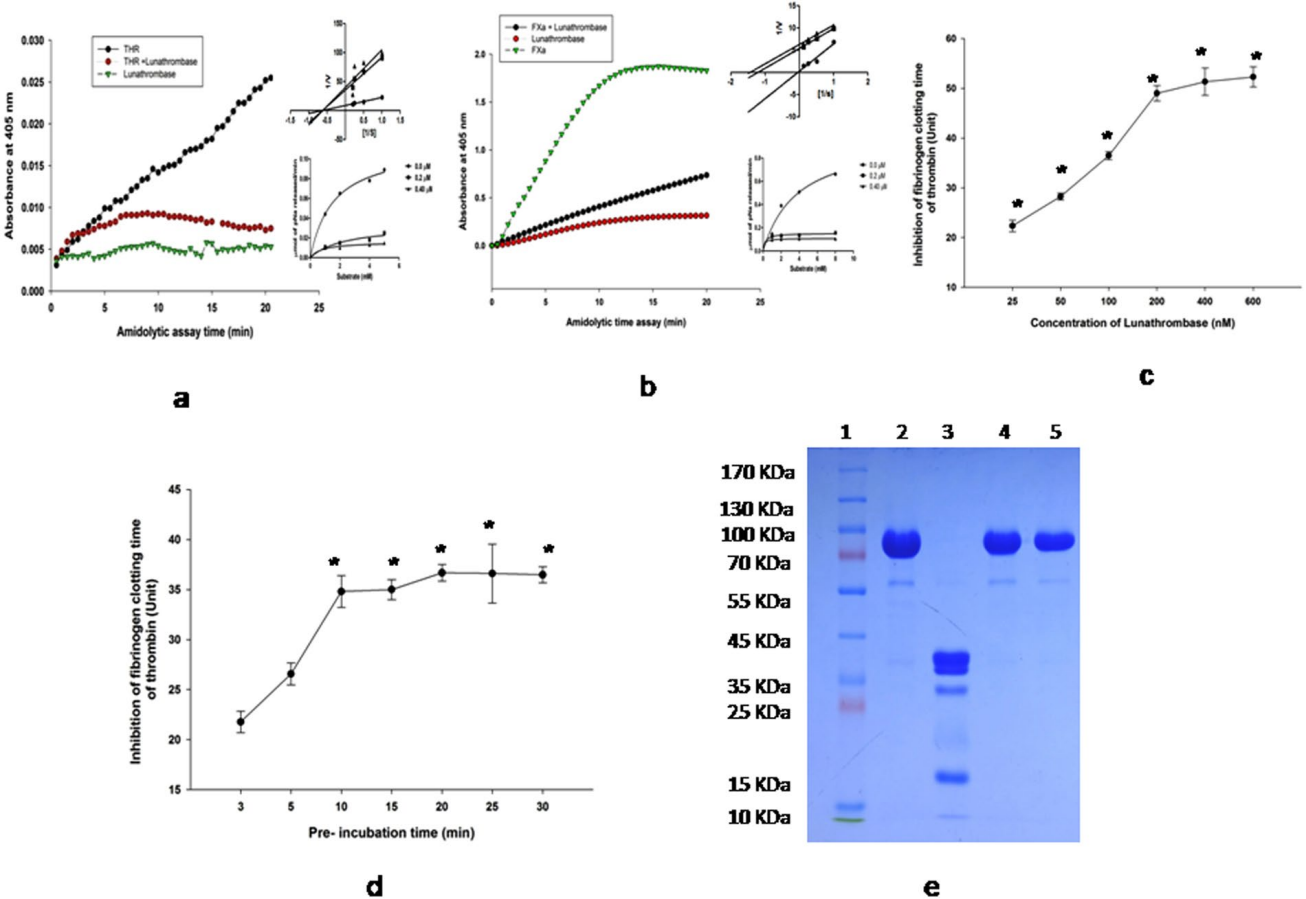
Effect of lunathrombase (0.2 µM) on amidolytic activity of (**a**) thrombin (36.6 nM) against its chromogenic substrate T1637 (0.2 mM). Inset. Michaelis-Menton and Lineweaver-Burk plot showing inhibition of amidolytic activity of thrombin towards T1637 (0.2 mM) by lunathrombase (0–0.4 µM). The plots are the means of 3 independent measurements. (**b**) FXa (0.13 µM) against its chromogenic substrate F3301 (0.2 mM). The values are mean of triplicate determinations. Inset. Michaelis-Menton and Lineweaver-Burk plot showing inhibition of amidolytic activity of FXa towards F3301 (0.2 mM) by lunathrombase (0–0.4 µM). The plots are the means of 3 independent measurements. (**c**) Inhibition of fibrinogen clotting activity of thrombin by lunathrombase (25–600 nM) at 37 °C, pH 7.4. (**d**) Time- dependent inhibition of fibrinogen clotting time of thrombin by lunathrombase (100 nM) at 37 °C, pH 7.4. The fibrinogen clotting time of thrombin under identical experimental conditions (control) was found to be 39.17 ± 1.54 s. The values are mean ± S.D. of triplicate determinations. Significance of difference with respect to control (without lunathrombase) *p < 0.05. (**e**) Inhibition of prothrombin activation property of FXa by lunathrombase. After reduction with β-mercaptoethanol, degradation products were separated by 12.5% SDS-PAGE. Lane 1, protein molecular markers; lane 2, 1.4 µM PTH; lane 3, PTH (1.4 µM) incubated with FXa (0.13 µM) for 30 min at 37 °C, pH 7.4; lane 4, [FXa (0.13 µM) pre-incubated with lunathrombase (0.2 µM) for 15 min] + PTH (1.4 µM); lane 5, PTH + lunathrombase.

Lunathrombase significantly inhibited the amidolytic activity of thrombin (Fig. 4a) and FXa (Fig. 4b). The *Ki* value for the inhibition of amidolytic activity of thrombin and FXa by lunathrombase was determined to be 26.90 ± 0.9 nM and 10.35 ± 1.8 nM (mean ± SD, n = 3), respectively. Lunathrombase dose-dependently prolonged the fibrinogen clotting time of thrombin and the saturated thrombin inhibition was observed at a 200 nM concentration of lunathrombase (Fig. 4c). The optimum inhibition was observed at 10 min of pre-incubation with thrombin and 200 nM lunathrombase (Fig. 4d). Further, lunathrombase (0.2 µM) completely (100%) inhibited the prothrombin activation by FXa (Fig. 4e). Nevertheless, SDS-PAGE analysis did not show thrombin degradation by lunathrombase (data not shown) suggesting that its anticoagulant mechanism does not depend on the catalytic degradation of thrombin.

### Spectrofluorometric analysis shows the interaction of lunathrombase with thrombin/fibrinogen/FXa

**Figure 5:**
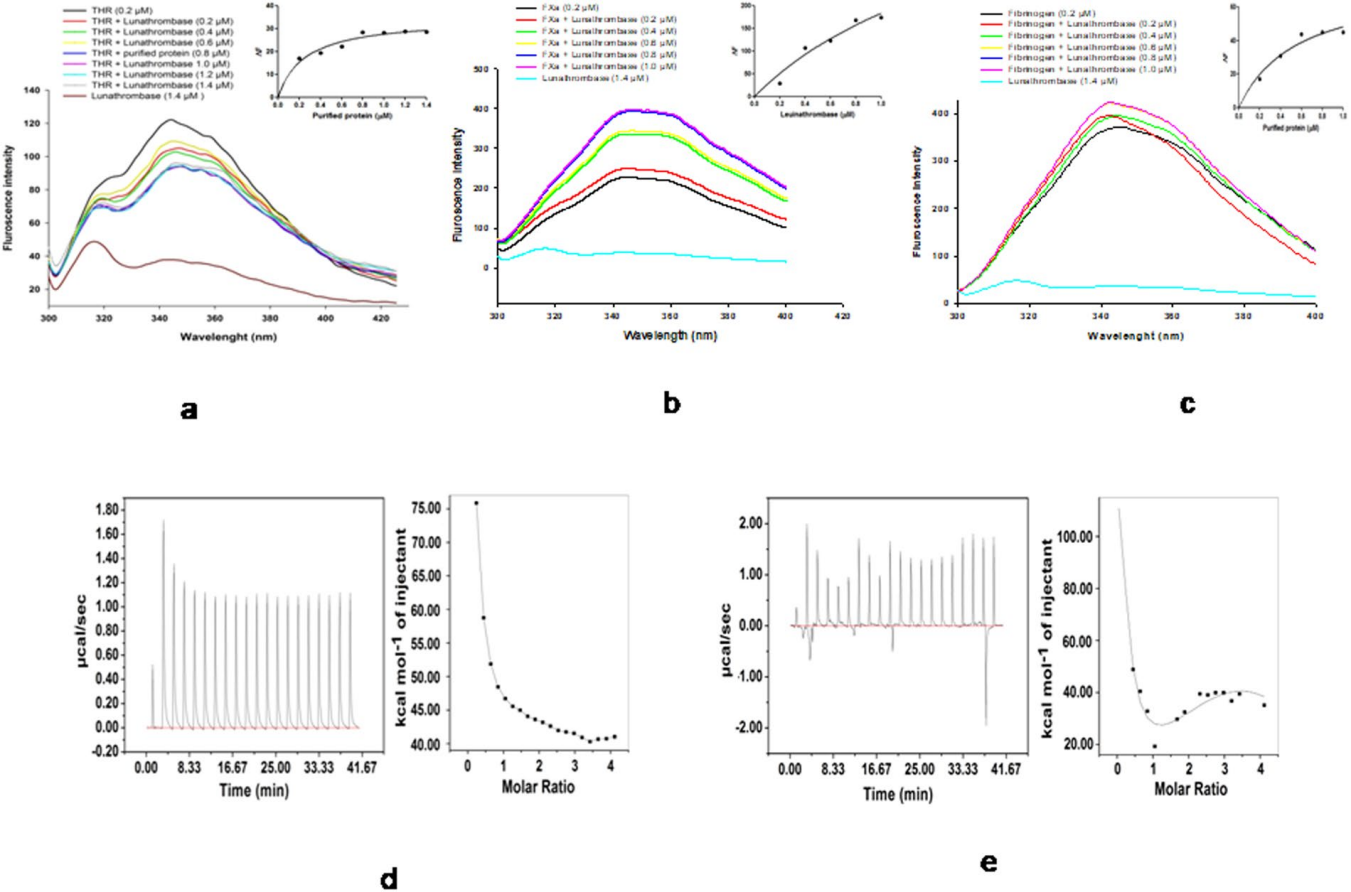
(**a**) Fluorescence spectra showing interaction of thrombin (0.2 µM) with different concentrations of lunathrombase (0.2–1.4 µM). Inset. One site saturation binding curve of lunathrombase for thrombin. (**b**) Fluorescence spectra showing interaction of FXa (0.2 µM) with different concentrations of lunathrombase (0.2–1.4 µM). Inset. One site saturation binding curve of lunathrombase for FXa. (**c**) Fluorescence spectra showing interaction of fibrinogen (0.2 µM) with different concentrations of lunathrombase (0.2–1.4 µM). Inset. One site saturation binding curve of lunathrombase for fibrinogen. (**d**) ITC profile for lunathrombase (10 µM) binding to thrombin (200 µM). Left panel shows heat change upon ligand addition; right panel shows an integrated ITC isotherm and is best fit to a sequential binding site model. (**e**) ITC profile for lunathrombase (10 µM) binding to fibrinogen (200 µM). Left panel shows heat change upon ligand addition; right panel shows an integrated ITC isotherm and is best fit to a sequential binding site model.

A steady decrease in the fluorescence intensity of thrombin was observed in the presence of lunathrombase (Fig. 5a). The interaction between lunathrombase and FXa (Fig. 5b) or fibrinogen (Fig. 5c) resulted in an increase in the fluorescence intensity, compared to the fluorescence intensity of individual proteins. The dissociation constant (*Kd*) for the binding of lunathrombase to thrombin, FXa, and fibrinogen was calculated to be 0.2492 µM, 1.908 µM, and 0.5516 µM, respectively (Fig. 5a–c).

### Isothermal calorimetry analysis of the interaction between lunathrombase and thrombin or fibrinogen

The titration of thrombin or fibrinogen with lunathrombase resulted in a strong endothermic generation of heat with a clear sigmoidal saturation curve indicating the direct binding interactions (Fig. 5d,e). The best fit for the titration curve was obtained with a sequential binding site model with a binding constant (*Ka*) of 9.7 × 10^−4^ M^−1^, ΔH = 2.07 × 10^−5^, ΔS = 717 cal/mol/deg for the interaction between lunathrombase and thrombin, and 1.02 × 10^−5^ M^−1^, ΔH = 2.3 × 10^−5^, ΔS = 816 cal/mol/deg for the interaction between lunathrombase and fibrinogen.

### Lunathrombase has *in vitro* thrombolytic potency but is devoid of hemolytic activity or cytotoxicity against mammalian cells

The *in vitro* thrombolytic potential of lunathrombase and commercial thrombolytic agents (streptokinase, plasmin, Nattokinase and tissue plasminogen activator) is shown in Supplementary Fig S14. The *in vitro* thrombolytic activity of equimolar concentrations of streptokinase, plasmin, Nattokinase, t-PA and lunathrombase was found to be identical. Nevertheless, the thrombolytic potency of lunathrombase, Nattokinase, and plasmin towards a heat-treated blood clot was reduced to 80%, 75%, and 60%, respectively of their original activity to dissolve an unheated blood clot (Supplementary Fig. S14). Streptokinase and tissue plasminogen activator showed negligible activity (<1%) in dissolving a heated blood clot (Supplementary Fig. S14).

Lunathrombase did not show *in vitro* rupturing of mammalian erythrocytes or cytotoxicity against HEK 293 cells (Supplementary Fig. S15). The fluorescence microscopic study indicated that at 24 h of treatment, lunathrombase did not change the cell morphology or membrane integrity of treated-HEK 293 cells in comparison to control cells (Supplementary Fig. S16). Further, no significance difference (p > 0.05) was found in G1, S, and G2 phases of lunathrombase-treated cell as compared to control HEK 293 cells (Supplementary Fig. S17).

### Lunathrombase showed antiplatelet activity by inhibiting collagen/ADP/arachidonic acid-induced platelet aggregation

**Figure 6:**
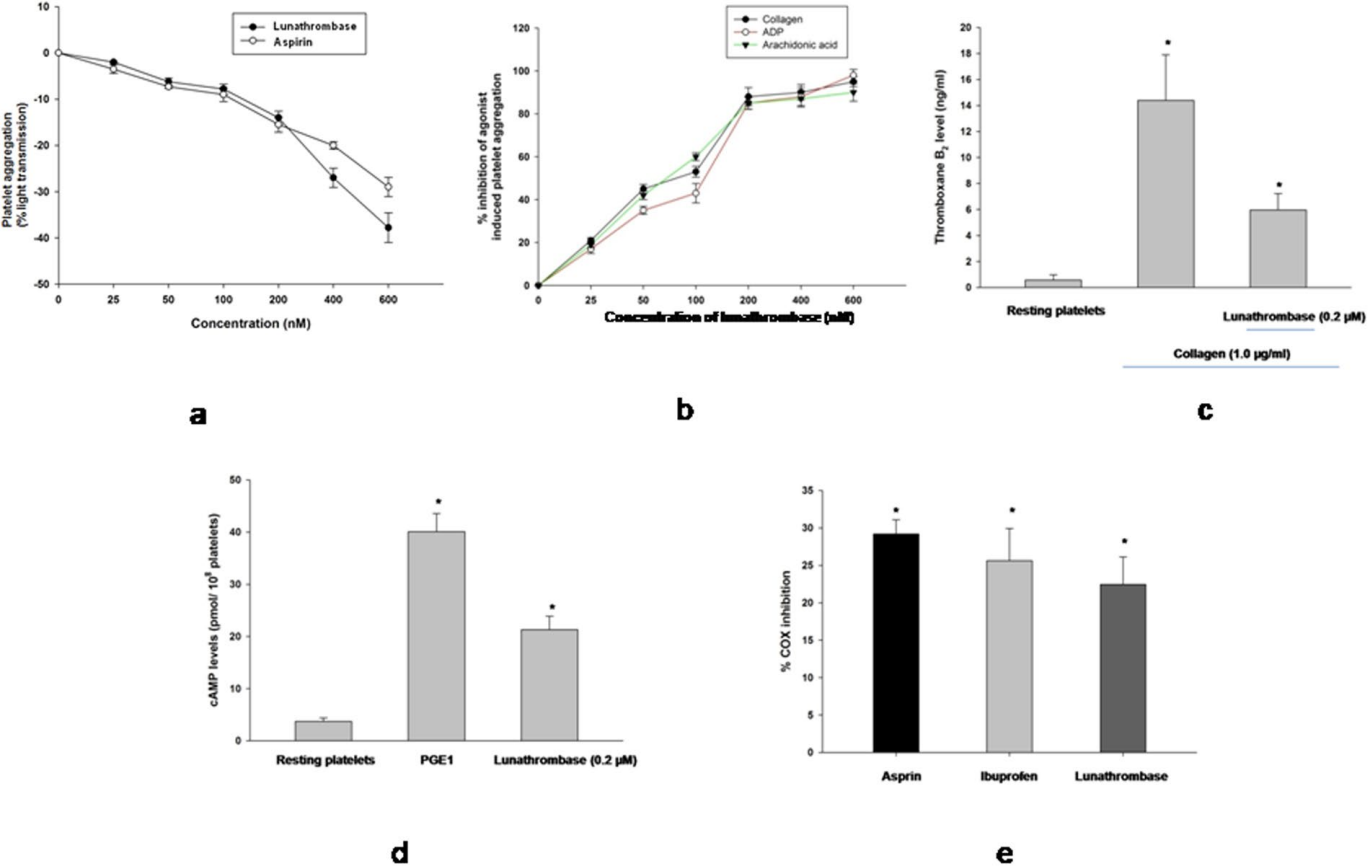
(**a**) Dose-dependent platelet deaggregation by lunathrombase / aspirin. Different concentrations of (0–600 nM) of lunathrombase or aspirin were incubated with platelet rich plasma at 37 °C and absorbance was recorded at 540 nm. Data represent mean ± SD of triplicate experiments. (**b**) Dose-dependent inhibition of collagen / ADP / arachidonic acid -induced platelet aggregation by lunathrombase. Different concentrations of lunathrombase (0–600 nM) was incubated with PRP at 37 °C for 10 min and then collagen (6.2 nM) / ADP (30 µM) / arachidonic acid (15 μM) was added in the reaction mixture. The percent platelet aggregation by collagen/ADP/arachidonic acid in absence of lunathrombase was considered as 100% activity and other values were compared to this. The IC_50_ value of lunathrombase (that showed 50% inhibition of collagen / ADP / arachidonic acid -induced platelet aggregation) was determined from the regression analysis of inhibition curve. Data represent mean ± SD of triplicate experiments. Effect of lunathrombase on (**c**) thromboxane B_2_ and (**d**) cAMP formation in activated platelets. Washed platelets were pre-incubated with lunathrombase (0.2 μM) or 0.5% DMSO on intraplatelet levels of cAMP formation in human platelets. Platelets were incubated with PGE1 (0.2 μM, positive control) or lunathrombase (0.2 μM) for measurement of cAMP formations. (**e**) Effect of lunathrombase on COX-1 activity. COX-1 enzyme was pre-incubated with lunathrombase or aspirin or ibuprofen (0.2 μM) for 30 min at 37 °C. The activity of control (without drugs) was considered as 100% and other values were compared with that. All values are means ± S.D. of triplicate determinations. Significance of difference with respect to control (without lunathrombase), *p < 0.05.

A comparable dose-dependent platelet de-aggregation (antiplatelet) property was displayed by equimolar concentrations of lunathrombase and aspirin (Fig. 6a). Lunathrombase also exhibited a dose-dependent inhibition of the collagen/ADP/arachidonic acid-induced aggregation of PRP (Fig. 6b). The concentration at which lunathrombase demonstrated 50% inhibition (IC_50_) of collagen/ADP/arachidonic acid-induced platelet aggregation was determined at 152.82 nM, 181.26 nM, and 159.89 nM, respectively (Fig. 6b).

The platelet deaggregation property (antiplatelet activity) of catalytically inactive lunathrombase was reduced to ~30% of its original activity exhibited by the catalytically active lunathrombase (Supplementary Fig. S18). However, there was no difference (p > 0.05) between the catalytically active and inactive lunathrombase in inhibiting the collagen/ADP/arachidonic acid-induced platelet aggregation (Supplementary Fig. S18). Fibrinogen induced aggregation of chymotrypsin-treated platelets but did not show aggregation of control (untreated) platelets (Supplementary Fig. S19). On the contrary, native and PMSF-treated lunathrombase caused deaggregation of α-chymotrypsin-treated as well as control platelets. Further, catalytically inactive lunathrombase did not interfere the binding of fibrinogen to platelet receptor GPIIb/IIIa (Supplementary Fig. S20).

### Lunathrombase increased cAMP level and inhibited COX-1 enzyme to exert its antiplatelet effect

Lunathrombase (0.2 µM) significantly inhibited the collagen (1 µg/ml)-stimulated TxB_2_ formation in washed platelets (Fig. 6c). Exogenous addition of lunathrombase (0.2 µM) to washed platelets increased its endogenous cAMP level (Fig. 6d). Furthermore, lunathrombase inhibited the COX-1 activity of collagen-treated platelets (Fig. 6e). In *in vitro* condition lunathrombase non-competitively inhibited the COX-1 enzyme with a *Ki* value of 5.947 ± 0.97 µM. The *Km and Vmax* values of lunathrombase towards COX-1 enzyme were determined at 1.5 ± 0.16 µM and 138.5 ± 5.7 µM/min (mean ± SD), respectively (Supplementary Fig. S21).

### Lunathrombase was non-toxic to rats but demonstrated *in vivo* anticoagulant and defibrino-genating activity

Lunathrombase at a dose of 10.0 mg/kg was found to be non-toxic to rats and showed no adverse effects or behavioral changes in treated-rats. The hematological parameters of blood from lunathrombase-treated rats (72 h post-treatment) did not show any significant deviation compared to the control group of rats; however, a minor increase in neutrophil content was found in the blood of the treated group of rats compared to control rats, which was within the normal range (Supplementary Table S4). In addition, none of the serum parameter of the treated rats was found to change (p > 0.05) in comparison to the serum profile of control group of rats (Supplementary Table S5). Plasma IgG, IgA, and IgE contents of lunathrombase-treated rats did not differ significantly from those of the control group of rats (data not shown). Light microscopic examination of the liver, kidney, and cardiac tissues of the lunathrombase-treated rats did not show any morphological alterations or pathophysiological symptoms (data not shown).

**Figure 7:**
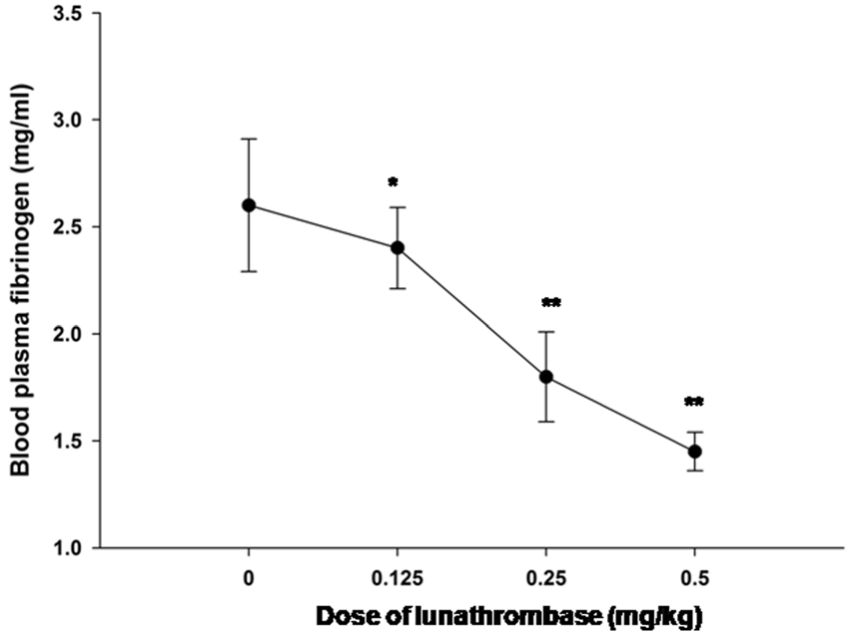
Dose- dependent *in vivo* defibrinogenating activity of lunathrombase 5 h after *i.p*. injection in rats. Values are means ± S.D. of triplicate determinations. Significance of difference with respect to control, *p < 0.05, **p < 0.01.

Lunathrombase demonstrated dose-dependent *in vivo* defibrinogenation of rat plasma (Fig. 7) with a corresponding dose-dependent increase in the *in vitro* tail bleeding time, Ca-clotting time and PT of PPP in the treated group of rats compared to the control group (Table 1).

**Table 1:** A comparison of *in vivo* anticoagulant activity of lunathrombase, heparin and Nattokinase treated Wister rats. The blood was withdrawn 5 h after *i.p*. injection of lunathrombase (0.5 mg/kg) or heparin (0.5 mg/kg) or Nattokinase (0.5 mg/kg).Values represent mean ± SD of six determinations. Significance of difference with respect to control. *p < 0.01. INR = (prothrombin_test_/prothrombin_control_).

	PT (s)	PT (INR)	APTT (s)	APTT (INR)	Tail bleeding time (s)	Plasma clotting time (s)
Control rats	14.84 ± 0.77	1.0	28.37 ± 1.4	1.0	45.33 ± 2.51	175.5 ± 9.14
Lunathrombase- treated rats (0.5 mg/kg)	28.1 ± 2.9*	1.89	30.23 ± 2.11	1.06	110 ± 1.41*	216.3 ± 8.8*
Heparin-treated rats (0.5 mg/kg)	21.48 ± 2.6*	1.44	32.77 ± 2.06	1.16	137.5 ± 4.9*	202.5 ± 10.3*
Nattokinase- treated rats (0.5 mg/kg)	20.1 ± 0.2*	1.35	30.33 ± 4.1	1.06	116.0 ± 8.0	201.3 ± 13.1*

## Discussion

The present study is the first to report on the purification and characterization of a fibrin(ogen)olytic serine protease showing strong anticoagulant, antithrombotic, and thrombolytic activities from the leaves of *L. indica*. The proteomics and amino acid composition analyses suggest that lunathrombase is a previously uncharacterized novel plant protease. Lunathrombase is an αβ-fibrinogenase, because it can degrade both α- and β-chains of fibrinogen^24–28^, demonstrating a fibrinogen degradation pattern that differs from other plant proteases^1,21,29–31^. The significant inhibition of the enzymatic activity of lunathrombase by serine protease inhibitors unambiguously demonstrates that lunathrombase is a serine protease and lack intramolecular and intermolecular disulfide linkage(s)^32–34^. Failure to inhibit the protease activity of lunathrombase by α_2_MG or antiplasmin suggests that this enzyme may also exert its activity *in vivo*.

Defibrinogenation, inhibition of platelet aggregation, and/or interference with components of the blood coagulation cascade are some of the key mechanisms by which proteolytic enzymes exert their anticoagulant effect^1,32,35^. The anticoagulant action of lunathrombase is due to its fibrinogenolytic property which is substantiated by its potency to inhibit thrombin and FXa as well as its antiplatelet effect. Spectrofluorometric analysis suggested the interactions between lunathrombase and thrombin/FXa/fibrinogen^36,37^. The higher *Kd* value indicated stronger interactions between lunathrombase and thrombin/FXa compared to those between lunathrombase and fibrinogen. Nevertheless, lunathrombase exerts its catalytic activity only on fibrinogen and would therefore inhibit thrombin/FXa by a non-enzymatic mechanism. The lower *km* value of lunathrombase towards fibrinogen, compared to Nattokinase and fibriongen indicates higher specificity of the former enzyme for the physiological substrate instead of Nattokinase.

The interaction between lunathrombase and thrombin/fibrinogen was also ascertained by ITC titration, which indicated a direct interaction between lunathrombase and thrombin/fibrinogen. The higher ΔH values suggest that the interactions are enthalpy-driven with the primary contributions to the complex stabilization likely resulting from electrostatic interactions and/or hydrogen bonds^38,39^. The *Kd* value is inversely proportional to the *Ka* value; the higher the *Ka* value for lunathrombase towards thrombin vs. fibrinogen indicates its higher affinity for thrombin, which is consistent with the spectrofluorometric analysis. The anticoagulant mechanism of lunathrombase appears to differ from that of the currently available anticoagulant drugs such as heparin and warfarin that act via the indirect inhibition of thrombin and by vitamin K antagonism, respectively^40,41^. The dual inhibition of thrombin and FXa by lunathrombase may lead to its consideration as an alternative new drug to the traditional cardiovascular drugs^15^.

A close association exists between platelet aggregation and the initiation of thrombus formation^42^. ADP or collagen, bind to the purinergic receptors P2Y1 and P2cyc, GPVI receptors, Integrin αIIbβ3, and the fibrinogen receptor on the platelet surface to induce platelet aggregation and adhesion^43,44^. The precise control of platelet function is an obligatory requirement for preventing thrombotic events^44–46^. The inhibition of platelet aggregation by lunathrombase was corroborated by a significant increase in the platelets cytosolic cAMP level. The activation of human platelets is inhibited by intracellular cAMP and cGMP-mediated pathways^47^. An increase in the intraplatelet levels of cAMP has been shown to downregulate the expression of the P2Y1R ADP-receptor, which is necessary for shape change^48^, maintaining GPVI (collagen receptor) in a monomeric form, keeping platelets in a resting state^49,50^, and inhibiting the release of sCD40L from platelets via the HSP27/p38 MAP kinase pathway^51^. The COX-1 isoenzyme is involved in the synthesis of prostaglandin that participates in platelet aggregation via the prostaglandin derivative, thromboxane B_2_^52,53^. The inhibition of COX-1 leads to inhibition of thromboxane B_2_ synthesis, which results in the inhibition of platelet aggregation^53^. Although *ex-vivo* study has shown the inhibition of COX-1 by lunathrombase; however, being a large molecule its direct interaction with intracellular COX-1 is unlikely. Therefore, it may inhibit COX-1 as well as up regulate the intracellular cAMP level by an indirect mechanism(s) which remains to be explored. Further, lunathrombase deaggregates platelets by both enzymatic and non-enzymatic mechanisms, though the latter mechanism predominates. However, the equipotent inhibition of collagen / ADP/ arachidonic acid-induced platelet aggregation by native and catalytically inactive lunathrombase indicates that these inhibitions are independent of the enzymatic activity of lunathrombase.

Limited proteolysis with α-chymotrypsin exposes glycoproteins GPIIb and GPIIIa, the two subunits of the platelet fibrinogen receptor GPIIb/IIIa complex at the surface of platelets, without interfering with cell activation or granular secretion^54^. Subsequently, fibrinogen binds to α-chymotrypsin-treated platelets to induce their aggregation^54^. Nevertheless, platelet deaggregation of α-chymotrypsin-treated platelets, caused by both native and PMSF-treated (catalytically inactive) lunathrombase suggests that this protease does not hydrolyze the fibrinogen receptor GPIIb/IIIa complex of platelets to exert the antiplatelet activity. Proteins harboring an RGD motif are shown to bind to GPIIb/IIIa platelet receptor and they may interfere the binding of fibrinogen to the receptor thereby inhibiting platelet aggregation^55,56^. Although presence of RGD motif in lunathrombase is unknown; however, this protease did not show binding to platelet GPIIb/IIIa receptor, and did not interfere the binding of fibrinogen to this receptor to inhibit platelet aggregation. This unequivocally indicates that the platelet deaggregation property (antiplatelet activity) of lunathrombase is not associated with impeding the binding of fibrinogen to platelet GPIIb/IIIa receptor. The exact mechanism of the anti-platelet activity of lunathrombase is our next goal of study. Further, the characterization of the clot bursting activity of lunathrombase provides a fair indication that lunathrombase is a plasmin-like, direct-acting fibrinolytic enzyme reinforcing its possible therapeutic application as a thrombolytic agent.

Administration of lunathrombase at a dose of 10.0 mg/kg, which is approximately 90 times greater than its anticoagulant dose (0.125 mg/kg), did not produce acute toxicity or adverse pharmacological effects in rats, indicating its preclinical safety and high therapeutic index. Hyperfibrinogenemia in blood is associated with increased risk of cardiovascular disorders^57^ and may promote the growth of lung and prostate cancer cells through interactions with fibroblast growth factor 2^58^. Lunathrombase also demonstrated *in vivo* defibrinogenation potential which leads us to anticipate the possible therapeutic applications of lunathrombase for treating and/or preventing cardiovascular diseases and the need for clinical trials.

## Conclusion

Ethnomedicines are regarded as depositories of potential therapeutic molecules that may affect the process of hemostasis. Lunathrombase is a previously uncharacterized non-toxic fibrinogenolytic from leaves of *L. indica*. It was characterized biochemically and pharmacologically and shown to exert dual inhibition of both thrombin and FXa via different non-enzymatic mechanisms. The potent *in vitro* and anticoagulant effects of lunathrombase suggest its pharmacological significance as an anticoagulant drug. It also showed *in vitro* antiplatelet and thrombolytic activities and *in vivo* defibrinogenating activity. In summary, therapeutic applications of lunathrombase for preventing and/or treating hyperfibrinogenemia- and thrombosis-associated cardiovascular disorders seem promising.

## Materials and Methods

### Chemicals

Coagulation proteins were purchased from Calbiochem, Germany. Human thrombin, prothrombin, human fibrinogen, and extracellular matrix (ECM) proteins like type-IV collagen, laminin, and fibronectin were purchased from Sigma Aldrich, USA. All commercially available drugs, like tissue plasminogen activator, streptokinase and plasmin were purchased from Sigma Aldrich, USA. Nattokinase was purchased from Healthy Origins, Pittsburg, USA. PT and APTT kits were purchased from Tulip diagnostics, Mumbai. LDL, HDL, and triglycerides assay kits were purchased from Diatek Healthcare Pvt. Ltd., Kolkata, India. The cholesterol assay kit was purchased from Sirus Biocare Pvt. Ltd., Kolkata, India, the fibrinogen assay kit was purchased from R^2^ Diagnostics, USA, and the immunoglobulin EIA kits were obtained from Thermo Fisher Scientific, USA. All other reagents were of analytical grade and purchased from Sigma Aldrich, USA.

### Collection of plant leaves and preparation of the aqueous extract

Leaves of *L. indica* were collected from 20 cm tall herbs, from areas surrounding the Sivasagar district, Assam (26.9844°N, 94.6314°E). The identity of the plant was confirmed by the Botanical Survey of India (BSI), Shillong, Meghalaya and a voucher specimen was deposited (accession number 37604). Fresh leaves of *L. indica* (100 g wet weight) were homogenized in a blender for 10 min and the extraction was carried out by stirring the crushed fresh leaves in ultrapure water (arium® advance EDI water purification system, Sartorius) (The pH of the water was adjusted to 7.4 by adding 0.01 N NaOH) for 4 h at 4 °C. The extract was filtered through muslin cloth followed by 0.45 µM pore-sized filter paper (Whatman, USA) and the filtrate was centrifuged (Multifuge X1R, Thermo Scientific) at 10,000 rpm for 10 min at 4 °C. The supernatant was collected, lyophilized, weighed, and stored at 4 °C until further use.

### Purification of the fibrinogenolytic protease (lunathrombase)

The dried extract (25.0 mg dry weight) was dissolved in 500 µl of 20 mM potassium phosphate buffer, pH 7.4, filtered through a 0.2 µm nylon syringe filter (Genetix Biotech Asia Pvt. Ltd.), and then loaded on a Hi Prep^TM^ anion- exchange column (pre-equilibrated with the above buffer) attached to a Fast Protein Liquid Chromatography (FPLC) system (AKTA purifier 10, Wipro-GE Healthcare Biosciences, Upsala, Sweden). After washing the unbound and the non-specifically bound proteins with two volumes of equilibration buffer, the bound proteins were eluted using a 0.1–1.0 M NaCl gradient at a flow rate of 1 ml/min at 4°C. Fractions of 2 ml were collected and the elution of protein was measured at 280 nm. The protein content, anticoagulant activity, and fibrino(geno)lytic activity of each peak were screened (see below).

The fractions showing the significant anticoagulant and fibrinogenolytic activity were pooled, concentrated using a lyophilizer and then fractionated through a CM-Cellulose cation-exchange column (20 mm × 60 mm) that had been pre-equilibrated with 20 mM potassium phosphate buffer, pH 7.4 Fractions were eluted using a 0.1M-1.0 M NaCl gradient at a flow rate of 0.5 ml/min. Fraction elutions were monitored at 280 nm and 2.0 ml of each fraction was collected. Each fraction was screened for protein content^59^, anticoagulant activity, and fibrino(geno)lytic activity (see below).

The fractions showing the highest anticoagulant and fibrinogenolytic activity were pooled, lyophilized, and dissolved in 100 µl of 20 mM sodium phosphate buffer, pH 7.4. The solution was filtered through a 0.2 µm syringe filter and fractionated on a Shodex KW-803 column (5 µm, 8 × 300 mm) pre-equilibrated with the same buffer containing 150 mM NaCl. Fractionation was carried out with an equilibration buffer at a flow rate of 0.5 ml/min at 4°C in a UHPLC system (Dionex Ultimate Mate 3000 RSLC, Dreieich, Germany). Fraction elutions were monitored at 280 nm and 1.0 ml fractions were collected. The protein content of the peak showing the highest anticoagulant and fibrinogenolytic activity was determined and selected for further study.

### Determination of purity and molecular mass of lunathrombase

The purity and molecular mass of the lunathrombase (20.0 µg) was determined by SDS-PAGE (12.5%) under reducing conditions^60^. Protein was visualized by staining with 0.1% Coomassie Brilliant Blue R-250 and destaining with methanol/acetic acid/water (40:10:50). The approximate molecular mass of lunathrombase was determined from a plot of log MW of standards vs. migration distance^32,33^. The purity of lunathrombase (5.0 µg) was also determined by MALDI-TOF mass spectrometric analysis (4800 plus, MDS SCIEX, Applied Biosystem) as previously described^61^.

### Determination of amino acid composition, secondary structure, and LC-MS/MS analysis of lunathrombase

For amino acid composition analysis, our previously elucidated procedure was followed^14^. The amino acid composition of lunathrombase was searched for in Swiss-prot and TrEMBL databases using the AAcompldent of Expert Protein Analysis System (ExPASy) software (http://www.expasy.org/tool/aacompident)^14,59^. The secondary structure of lunathrombase was determined by measuring the circular dichroism (CD) spectrum (JASC0 J-815 CD Spectrometer) as described previously^60^. Yang’s reference was set for the CD analysis. CDPRO CLUSTER software was used to determine the secondary structure of lunathrombase^14^.

For the LC-MS/MS analysis, lunathrombase was in-gel trypsin digested following our previously described procedure^62^. The LC-MS/MS analysis of extracted tryptic peptides was done as we described previously^62,63^. The data was used to search for the identification of protein on the MASCOT 2.4 search engine against Swiss-Prot, TrEMBL, and non-redundant protein sequence databases from NCBI and the data were analyzed in Proteome Discoverer 1.3 software (ThermoFisher Scientific, Germany). A minimum of two high confidence peptides were used as a prerequisite to identify the protein. The *de novo* (independent database) sequencing with an average local confidence (ALC) score of ≥50% was derived directly from the MS/MS spectrum using PEAKS 7.0 software. The identified peptides were subjected to a BLAST search in NCBInr for Swissprot protein sequences (swissprot) and Protein Databank Proteins (PDB) against Lamiaceae family proteins, green plant proteins and all NCBI databases using the blastp algorithm (http://blast.ncbi.nlm.nih.gov/Blast.cgi).

### Protease assay

Human fibrinogen (2.6 µM)/human fibrin (Sigma–Aldrich, USA) (dissolved in 1x PBS, pH 7.4) was incubated with lunathrombase/Nattokinase/streptokinase/plasmin/thrombin (0.2 µM) from 15 to 120 min at 37 °C. The reaction was terminated by adding 20 µl of 6x SDS-PAGE Loading dye containing 3 mM β-mercaptoethanol and the tubes were heated at 100 °C (Digital block heater, Select Bioproducts) for 5 min. A control was run in parallel where 1x PBS, pH 7.4 was added. The digested fibrinogen/fibrin was separated by 12.5% SDS-PAGE at 120 V, and the protein bands were visualized by staining with Coomassie Brilliant Blue R-250 and destaining with methanol/acetic acid/water (40:10:50)^33^. The gel was scanned and analyzed by ImageJ software (version 1.47) (Wayne Rasband, NIH, USA) to calculate the percent degradation of α- and β-chains of fibrinogen/fibrin by lunathrombase/ Nattokinase/streptokinase/plasmin/thrombin considering the band intensity of these chains in the untreated (control) fibrinogen as 100%.

In another set of experiments, the human fibrinogen solution (2.5 mg/ml in 20 mM potassium phosphate buffer containing 100 mM NaCl, pH 7.4) was incubated with 0.2 µM of either lunathrombase or Nattokinase (commercial anticoagulant) for 15 min at 37 °C. The resulting supernatant was filtered through a 0.2 μm membrane filter and the fibrinogen degradation products were separated on a RP-UHPLC (Dionex Ultimate Mate 3000RSLC, Dreieich, Germany) Acclaim^®^ 300 C18 column (2.1 mm × 150 mm, 3 µm, 300 Å) as described previously^33^. From the standard curve of human fibrinopeptides A and B (Sigma-Aldrich, USA) eluted from the RP-HPLC column under identical conditions, the amount of lunathrombase-induced release of fibrinopeptides A and B from human fibrinogen was determined. A control was also run in parallel where fibrinogen solution alone was incubated with 0.1 ml of 1x PBS, pH 7.4^33^.

### Biochemical characterization

The optimum conditions for fibrin(ogen)olytic activity were determined by incubating 0.2 µM of lunathrombase with human fibrinogen (2.6 µM) (Sigma Aldrich, USA) at different pH (from 2–12) and temperatures (from 10–80 °C) followed by measuring the fibrin(ogen)olytic activity^32^.

The activity of lunathrombase against other blood proteins (bovine serum albumin and bovine serum γ-globulin) was determined by incubating 0.2 µM of enzyme with 2.6 µM of substrate dissolved in 20 mM potassium phosphate buffer, pH 7.4 at 37 °C for 30 min. Protease activity was determined by the colorimetric method, as described previously^32^. One unit of protease activity was defined as 1 µg of tyrosine liberated per min per ml of enzyme^32^.

Esterolytic activity was assayed by the spectrophometric method using N_α_-*p*-Tosyl-L- arginine methyl ester hydrochloride (TAME) and N_α_-Benzoyl-L- arginine ethyl ester hydrochloride (BAEE) as substrates. TAME esterase activity (in 50 mM Tris-HCl, 100 mM KCl, pH 8.1) was determined as described by Costa *et al*.^64^. One unit of TAME-esterase activity is defined as an increase in absorbance of 0.01 at 244 nm during the first 10 min of the reaction at 37 °C. For BAEE-esterase activity, the protocol described by Rutkowski^65^ was followed. The assay was carried out in 100 mM Tris-HCl, pH 8.0 at 37 °C for 10 min. One unit of BAEE-esterase activity is defined as an increase in absorbance of 0.01 at 254 nm during the first 5 min of the reaction at 37 °C. For every experiment, a control was run in parallel instead of lunathrombase, using an equivalent volume of buffer. Activity was expressed as units of TAME or BAEE/mg of lunathrombase.

The *Km* and *Vmax* values of lunathrombase against fibrinogen were determined by incubating a fixed concentration (0.2 µM) of protease under study or Nattokinase with different concentrations of fibrinogen (1 to 6 µM) at 37 °C for 60 min and the protease activity at each concentration of substrate was determined. The kinetic parameters of lunathrombase and Nattokinase were determined by nonlinear regression analysis using GraphPad Prism 5.0 software^12^.

The effect of lunathrombase on extracellular matrix (ECM) proteins such as laminin, type-IV collagen, and fibronectin was determined by incubating lunathrombase with substrate at a 15:1 ratio (w/w), in a total volume of 20.0 µl of 1x PBS buffer, pH 7.4 at 37 °C for 12 h. The reaction was stopped immediately after the stipulated time interval by chilling in ice and adding 5.0 µl denaturing buffer containing SDS and β-mercaptoethanol. The degradation products were analyzed on 10% SDS-PAGE after staining with 0.25% Coomassie Brilliant Blue R-250.

The influence of metal ions (Cu^2+^, Co^2+^, Ca^2+^, Zn^2+^, Mg^2+^, Mn^2+^, and Fe^2+^) was determined by pre-incubating the lunathrombase (0.2 µM) with the respective metal ions (2.0 mM at final concentration in 1x PBS buffer, pH 7.4) for 30 min at 37 °C and then assaying the fibrin(ogen)olytic activity. Irreversible chemical modification of the histidine, cysteine, and serine residues was performed by pre-incubating the lunathrombase (0.2 µM) with 4-bromophenacyl bromide (pBPB), iodoacetamide (IAA), and phenylmethylsulfonyl fluoride (PMSF) at final inhibitor concentrations of 2 mM and 4 mM at room temperature for 60 min and then assaying the protease activity^14,32^. For disulfide bond reduction and metalloprotease activity, lunathrombase was treated with DTT and EDTA, respectively (2 mM and 4 mM) at 37 °C for 60 min.

In another set of experiments, lunathrombase (0.2 µM) was pre-incubated with α_2_-macroglobulin or antiplasmin (3.0 µM) for 60 min at 37 °C and the fibrin(ogen)olytic activity was then determined as described above. For every experiment, a control was run where the protease was incubated with an equivalent volume of assay buffer. The activity of the control was considered as 100% and the other values were compared to that^14,32^.

The total neutral sugar in lunathrombase was determined following the phenol-sulfuric colorimetric method^66^. From the standard curve of glucose, the amount of neutral sugar was determined. To determine the extent of N-linked or 0-linked oligosaccharides, lunathrombase was treated with PNGase and neuraminidase, respectively, following the instructions of the manufacturer (New England Biolabs, Inc., Ipswich, MA). Briefly, after denaturing lunathrombase at 100 °C for 10 min, the reaction was incubated with PNGase or neuraminidase for 4 h at 37 °C and the reaction products were separated by 12.5% SDS-PAGE under a reducing condition^33^. Native (untreated) and denatured lunathrombase were used as controls. The gel was stained with Commassie Brilliant Blue R-250 and destained with methanol/ acetic acid/water (40:10:50) to visualize the protein bands.

### Assay of anticoagulant activity and hemolytic property of lunathrombase

Goat blood obtained from a slaughterhouse was collected in 3.8% tri-sodium citrate and platelet poor plasma (PPP) and prepared according to our previously described protocol^62,67^. Different concentrations (50–800 nM) of lunathrombase were pre-incubated with 300 µL of PPP for 3 min at 37 °C, and clotting was initiated by adding 40 µL of 250 mM CaCl_2_^62^. For controls, instead of lunathrombase, the same volume of 1x PBS, pH 7.4 was used. One unit of anticoagulant activity of lunathrombase was defined as 1 s increase in clotting time for the control PPP^62,68^.

The activated partial thromboplastin time (APTT) and prothrombin time (PT) of lunathrombase-treated and control (untreated) PPP were measured using commercial kits^62^. The hemolytic activity of lunathrombase was determined against mammalian washed erythrocytes, as described by Doley *et al*.^69^.

### Thrombin and FXa inhibitory effect of lunathrombase and determination of the inhibitory constant (*Ki*)

Different concentrations of lunathrombase (25–600 nM) or 1x PBS, pH 7.4 (control) were pre-incubated with thrombin (3 µl, 10 NIH U/ml in 20 mM potassium phosphate buffer, pH 7.4) for 30 min at 37 °C. The reaction was started by adding 2.6 µM human fibrinogen (dissolved in 20 mM potassium phosphate buffer, pH 7.4) and the time of fibrin clot formation was monitored by visual inspection^12,33^.

For the FXa inhibition assay, lunathrombase (0.2 µM)/1x PBS (control) was pre-incubated with FXa (0.13 μM) in 20 mM sodium phosphate buffer, pH 7.4 at 37 °C for 30 min. Thereafter, 1.4 µM prothrombin (the physiological substrate for FXa) was added and the reaction mixture was incubated at 37 °C for 1 h. The prothrombin degradation products were analyzed by 12.5% SDS-PAGE under reducing conditions^12^.

The inhibition of the amidolytic activity of thrombin (3.0 µl, 10 NIH/ml) or FXa (0.13 µM) by lunathrombase (0.2 µM) was determined as described previously^12,14^. For the kinetics analysis, the reaction rate (V) was plotted against the substrate concentration (S) at each inhibitor concentration, and the data was fitted to a hyperbolic Michaelis - Menten model using GraphPad Prism 5.0 software^12^. The inhibitory constant (*Ki*) was determined using the competitive and non-competitive model for enzyme inhibition for thrombin and FXa, respectively using the above software^12^.

### Determination of interaction between lunathrombase and thrombin/fibrinogen/FXa by spectrofluorometric analysis

Thrombin (0.2 µM), fibrinogen (0.2 µM) and FXa (0.2 µM) were each incubated with different concentrations of lunathrombase (0.2 µM-1.0 µM) for 3 min at room temperature. The fluorescence intensity of the reaction mixture was monitored (excitation wavelength = 280 nm) by recording the emission spectrum in the range between 300 and 425 nm using a fluorescence spectrometer (LS55, Perkin Elmer) as we described previously^12,14^. The dissociation constant (*Kd*) for the binding of lunathrombase with thrombin/fibrinogen/FXa was determined as described previously^12,14^.

### Isothermal titration calorimetry titration to determine the interaction of lunathrombase with thrombin and fibrinogen

ITC experiments were performed at 37 °C on a MicroCal^TM^ iTC-200 system (GE Healthcare) in a high gain mode at a reference power of 10 µcals^−1^. Human thrombin or fibrinogen (200 µM in 1x PBS buffer, pH 7.4) was titrated against 10 µM of lunathrombase dissolved in the same buffer. A total of 20 injections were made with 300 s time intervals in between^70^. For longer titrations, the syringe was refilled and injections continued into the same cell sample. Control runs were performed in which cell samples and syringe samples were titrated with buffer and the data from these runs was subtracted from the experimental data. Data analysis was performed with Origin software whereas data fitting was done using a “sequential binding” model.

### Determination of *in vitro* thrombolytic activity

For the *in vitro* thrombolytic activity assay, lunathrombase or commercial thrombolytic agents such as s tissue plasminogen activator (tPA)/ streptokinase (indirect thrombolytic agent)/ plasmin (direct thrombolytic agent)/ Nattokinase (fibrinolytic agent) at a final concentration of 1.0 µM or 1x PBS buffer, pH 7.4 (control) was incubated with a mammalian (goat) blood clot for 3 h at 37 °C. The thrombolytic activity was determined as described by Majumdar *et al*.^14^. The *in vitro* thrombolytic activity was expressed as mg of blood clot (thrombus) lysed per μM of lunathrombase/commercial thrombolytic agents, compared to the control^14^. In another set of experiments, the blood clot was heated at 80 °C for 30 min to denature the endogenous fibrin(ogen)olytic factors (plasmin, plasminogen etc.) prior to the thrombolytic activity assay^14^.

### Antiplatelet effect of lunathrombase against collagen/ADP/arachidonic-induced platelet aggregation

The collection of blood from healthy volunteers (who were not under medication) was approved by the Tezpur University Ethical Committee and informed consent was obtained from all participants. Platelet rich plasma (PRP) was prepared from citrated human blood, following the procedure described previously^62,71^. Lunathrombase/aspirin (0–600 nM) was added to 100 μl of the PRP and the absorbance was measured continuously at 540 nm for 5 min, as stated above. The percent platelet aggregation after 300 s of incubation of platelets with agonists was calculated as described previously^62^.

In another set of experiments, PRP was pre-incubated with lunathrombase (0–600 nM) for 5 min prior to the addition of collagen (6.2 nM)/ADP (30 μM)/arachidonic acid (15 μM). The aggregation induced by the identical concentration of collagen/ADP/arachidonic acid was considered to be 100% activity and the decrease in lunathrombase-induced platelet aggregation (antiplatelet activity) was compared to that^62^.

### Effect of catalytically inactive lunathrombase on washed platelet and collagen/ADP/arachidonic acid-induced platelet aggregation

To inhibit the catalytic activity of lunathrombase, it was incubated with PMSF (4 mM) at 37 °C for 60 min. Thereafter, excess PMSF was removed by a Nanosep 3 K Omega membrane filter (Pall Corporation, USA) and the PMSF-inactivated enzyme was assayed for its catalytic inactivation by the protease assay. The effect of catalytically inactive lunathrombase (0.2 µM) on washed platelets and collagen / ADP / arachidonic acid-induced platelet aggregation was assayed as described above^62^. The activity of native lunathrombase was considered as 100% and other values were compared to that.

### Effect of lunathrombase on α-chymotrypsin-treated platelets

Washed platelets (1 × 10^6^) were treated with freshly prepared α–chymotrypsin (8 U/ml) for 15 min at room temperature and then centrifuged at 1500 × g for 15 min^72^. The pellet containing the platelets was washed with Ca^2+^- free Tyrode’s buffer (5 mM HEPES, 137 mM NaCl, 2.7 mM KCl, 12 mM NaHCO_3_, 0.42 mM Na_2_HPO_4_, 1 mM MgCl_2_, 0.1% glucose, and 0.25% bovine serum albumin) three times and then suspended in the same buffer. The platelet aggregation was initiated by adding human fibrinogen (0.2 µM)/lunathrombase (0.2 µM)/catalytically inactive lunathrombase (0.2 µM) to the chymotrypsin-treated or control platelet suspension. As a control, BSA (0.2 µM) was also added to the chymotrypsin-treated platelet to determine the platelet aggregation.

### Determination of binding of catalytically-inactivated lunathrombase with human GPIIb/IIIa and fibrinogen by ELISA

The binding of PMSF-inactivated Lunathrombase to human GPIIb/IIIa and fibrinogen was assessed by ELISA as described earlier^73,74^. Briefly, human fibrinogen (1000 ng) was coated to the wells of Nunc ELISA plates. After washing the unbound proteins by 1x PBS, pH 7.4 and blocking the wells with 5% fat-free milk in 1x PBS, pH 7.4, the wells were incubated with graded concentrations (0.2–1.0 µM) of PMSF-inactivated lunathrombase or 1x PBS (control) and incubated at room temperature for 2 h. Thereafter, human GPIIb/IIIa (500 ng) was added to the wells and further incubated for 2 h at room temperature. Thereafter, mouse anti-GPIIb/IIIa antibody (1: 1000 dilutions) was added to wells, incubated for 2 h, washed with 1x PBS, pH 7.4 for three times. Rabbit anti-mouse IgG-HRP conjugated secondary antibody (1:2000 dilutions) was incubated for 2 h at room temperature to detect the primary antibody. Color was developed by adding substrate (1x TMB/H_2_O_2_) to the well for 30 minutes in dark condition and reaction was stopped by adding 50 µl of 2 M H_2_SO_4._ The absorbance was taken at 492 nm against blanks in Multiskan GO (Thermoscientific, USA) microplate reader. The binding of GPIIb/IIIa to control fibrinogen was considered as 100% binding and other values were compared to that.

In another set of experiments, PMSF-inactivated lunathrombase (0.2–1.0 µM) or 1x PBS, pH 7.4 (control) was added to the human GPIIb/IIIa (500 ng) coated wells, incubated at room temperature for 2 h and washed with 1x PBS. Then, anti-GPIIb/IIIa antibody (1: 1000) was added, incubated at room temperature for 2 h, washed with 1x PBS and then HRP-conjugated anti-mouse IgG (1:2000) was added to wells and incubated for 2 h at room temperature to detect the primary antibody. The absorbance was taken at 492 nm against blanks.

### Determination of cAMP, thromboxane level of platelets, and COX-1 inhibitory effect of lunathrombase

Platelet suspensions (1 × 10^8^ platelets/ml) were pre-incubated with lunathrombase (0.2 µM) for 5 min and thereafter 2 mM EDTA and 50 µl indomethacin (1 mM) were added to the suspensions. The thromboxane B_2_ level (TxB_2_) and cAMP levels of the supernatants were measured by an enzyme immuno assay (EIA) kit (R&D systems, USA) following the instructions of the manufacturer. The cAMP level in the platelet suspensions incubated with 0.2 µM PGE1 (positive control) was also measured under identical conditions. The cyclooxygenase-1 (COX-1) inhibitory effect of lunathrombase/ibuprofen/aspirin (0.2 µM) was determined using the commercial kit (R&D systems, USA) following the instructions of the manufacturer.

### Cytotoxicity assessment of lunathrombase

The cytotoxicity of lunathrombase was tested against human embryonic kidney cells (HEK 293 cell line), cultured and maintained in Dulbecco’s modified eagle medium (DMEM) as described previously^75,76^. For the viability assay, 100 µl aliquots of 2 × 10^4^ cells/ml were seeded into 96-well plates and treated with various concentrations (0.2 to 2.0 µM) of lunathrombase dissolved in 1x PBS, pH 7.4 and medium (control), and incubated at 37 °C for 24 h. Cell viability was then determined using the colorimetric MTT [3-(4,5-dimethylthiazol-2-yl)-2,5-diphenyltetrazolium bromide] assay following the manufacturer’s instructions (ATCC)^73,74^. All assays were done in triplicate and repeated at least three times.

### Calcein-AM cell viability staining

HEK 293 cells (2 × 10^4^ cells/well), cultured in 96-well plates in DMEM media supplemented with 10% FBS, were treated with lunathrombase (2.0 μM) or medium (control) for 24 h. The cells were washed with 1x PBS, pH 7.4, stained with 5.0 μM calcein-AM (in 1x PBS, pH 7.4) and incubated for 5 min. The cells were washed with 1x PBS, pH 7.4, and visualized using an epi-fluorescence microscope at 40× magnification (Nikon ECLIPSE Ti-U, Tokyo, Japan)^77^. For the phase-contrast images, photomicrographs were captured using a Nikon ECLIPSE Ti-U (Tokyo, Japan) camera without filter.

### Flow cytometric analysis to determine the cell cycle kinetics

The effect of lunathrombase on the cell cycle of HEK 293 cells was determined by flow cytometry analysis using propidium iodide (PI) DNA staining dye^77,78^. HEK 293 cells (1.5 × 10^5^ cells per ml) were seeded in 96-well plates and allowed to adhere overnight at 37 °C. On the next day, the old medium was replaced with fresh media containing lunathrombase (2.0 μM) or only growth medium (control) and incubated for 24 h at 37 °C. Cells were collected by trypsinization and then fixed by adding chilled 70% ethanol before being stored at −20 °C until further analysis.

The fixed cells were centrifuged and washed with chilled 1x PBS, pH 7.4, following incubation with RNase at 37 °C for 1 h. Cells were then incubated with PI stain for 2 h before being analyzed by flow cytometry (FACscan, Becton Dickinson, Bedford, MA). The data was analyzed by ModFiT LT software.

### Determination of *in vivo* anticoagulant activity, defibrinogenating activity, and toxicity in an animal model

Acute *in vivo* toxicity of lunathrombase was evaluated in Wistar strain rats, using the protocol of the OECD/OCED guidelines 425 (2001). Animal experiment protocols were approved by the Tezpur University Animal Ethical Committee (Approval no: DORDPro/TUAEC/10–56/14/Res-10) and the Institute of Advanced Study in Science and Technology, Guwahati (Approval no: IASST/IAEC/2016-17/04).

For *in vivo* toxicity assessment, lunathrombase was dissolved in 0.2 ml of 1x PBS, pH 7.4, and injected (10 mg/kg body weight, *i.p*) into the albino Wistar strain rats (n = 6) weighing between 120–150 g. The control group of rats received only 0.2 ml of PBS, pH 7.4 (placebo). The treated rats were observed at regular intervals up to 72 h post-injection for death or any physical or behavioral change^75^. After 72 h of treatment, the rats were sacrificed and blood was collected immediately by cardiac puncture. Hematological parameters of the blood were analyzed by a Hematology Auto Analyzer MS4-S (Melet Schloesing Laboratories, Osny, France). Plasma was obtained from control and the treated groups of rats and analyzed for total protein, glucose, serum glutamic oxaloacetic transaminase (SGOT), serum glutamic pyruvic transaminase (SGPT), alkaline phosphatase (ALP), cholesterol, triglyceride, urea, and uric acid, using commercial diagnostic kits following the manufacturers’ instructions. All biochemical parameters were analyzed on an auto analyzer (Biochemical Systems International SRL Model 3000 evolution, Florence, Italy). Serum levels of immunoglobulins (IgA, IgE, IgG, and IgM) were determined by ELISA, using commercial kits and following the instructions of the manufacturer (Thermo Fisher Scientific, USA).

To examine possible lunathrombase-induced morphological alterations, the heart, liver, and kidney of the treated and control groups of rats were dissected. Tissues were cut into small pieces, washed with PBS, pH 7.2, to remove the adherent blood, and then placed in 10% buffered formaldehyde. The fixed tissues were dehydrated in a graded series of alcohol, embedded in paraffin, and processed routinely for light microscopic observation after hematoxylin-eosin staining^33^.

To determine the *in vivo* defibrinogenating and anticoagulant activities, different doses of lunathrombase (0.125, 0.25, and 0.5 mg/kg body weight of rats)/heparin (0.5 mg/kg)/Nattokinase (0.5 mg/kg) were injected (*i.p*) in Wistar strain rats (n = 6) and blood was withdrawn 5 h after injection by retro orbital bleeding. The PT, APTT, tail bleeding time, and plasma Ca-clotting time of control and treated groups of rats were determined as described previously^35,58,79^. The fibrinogen content of plasma was measured using commercial kits following the manufacturer’s protocol. At the end of the experiments, rats were euthanized with an overdose of Na-pentobarbital as per recommendations of the CPCSEA.

All experiments were carried out in accordance with the guidelines of the Tezpur University Ethical Committee, Institute of Advanced Study in Science and Technology, Guwahati and the bio-safety committee guidelines, Tezpur University.

### Statistical analysis

The statistical analysis of the data was done by Student’s t-test using the SigmaPlot 10.0 for Windows (version 7.0) software. A value of p < 0.05 was considered as a significant difference.

## Electronic supplementary material

Supplementary Figures and Tables
