# Supplementary material for: Anticoagulant mechanism, pharmacological activity, and assessment of preclinical safety of a novel fibrin(ogen)olytic serine protease from leaves of Leucas indica
Source: Sci Rep. 2018 Apr 18;8:6210. doi: 10.1038/s41598-018-24422-y (PMC5906637; doi:10.1038/s41598-018-24422-y)
Supplement: Supplementary file 1 — Supplementary Figures and Tables [file 41598_2018_24422_MOESM1_ESM.doc]

**Supplementary info**

**Anticoagulant mechanism, pharmacological activity, and assessment of preclinical safety of a novel fibrin(ogen)olytic serine protease from leaves of *Leucas indica***

Debananda Gogoi^1^, Neha Arora^2^, Bhargab Kalita^1^, Rahul Sarma^3^, Taufikul Islam^1^, Sidhhartha S. Ghosh^2^, Rajlakshmi Devi^3^, Ashis K. Mukherjee^1^*

^1^ Microbial Biotechnology and Protein Research Laboratory, Department of Molecular Biology and Biotechnology, School of Sciences, Tezpur University, Tezpur- 784028, Assam, India

^2^. Department of Biosciences and Bioengineering, Indian Institute of Technology, Guwahati-781039, Assam, India

^3^**.** Biochemistry Laboratory, Life Sciences Division, Institute of Advanced Study in Science and Technology, Guwahati-781035, Assam, India

***Corresponding Author:** Dr. A.K. Mukherjee, Department of Molecular Biology and Biotechnology, School of Sciences, Tezpur University, Tezpur- 784028, Assam, India.

Tel: +917896003886; E-mail address: [akm@tezu.ernet.in](mailto:akm@tezu.ernet.in)

**Contents:**

1. Supplementary figures S1-22

2. Supplementary tables S1-S5

**Supplementary Fig. S1.**  Multiple sequence alignment of tryptic fragment of lunathrombase with homologous proteins deposited in the NCBI database. 1H9H/E, chain E, complex of Eeti-Ii with porcine trypsin; NP_001156363, trypsinogen precursor [*Sus scrofa*]; 1EPT/B, chain B, refined 1.8 angstroms resolution crystal structure of porcine epsilon-trypsin; XP_020934119.1, trypsinogen isoform X1 [*Sus scrofa*] ; P00761.1, full=trypsin, flags: precursor.

1H9H|E -----------------------IVGGYTCSAANSIPYQVSLNSGSHFCSGGSLINSQWV

NP_001156363.1 MNTFVLLALLGAAVAFPTDDDDKIVGGYTCA-ANSVPYQVSLNSGSHFCG-GSLINSQWV

1EPT|B ------------------------------------------------------------

XP_020934119.1 MNTFVLLALLGAAVAFPTDDDDKIVGGYTCA-ANSIPYQVSLNSGSHFCG-GSLINSQWV

P00761.1 ---------------FPTDDDDKIVGGYTCA-ANSIPYQVSLNSGSHFCG-GSLINSQWV

Lunathrombase ------------------------------------------------------------

1H9H|E VSAAHCSYKSRIQVRLGEHNIDVLEGNEQFINAAKIITHPNFNGNTLDNDIMLIKLSSPA

NP_001156363.1 VSAAHC-YKSRIQVRLGEHNIDVLEGNEQFINAAKIITHPNFNGNTLDNDIMLIKLSSPA

1EPT|B ---------SRIQVRLGEHNIDVLEGNEQFINAAKIITHPNFNGNTLDNDIMLIKLSSPA

XP_020934119.1 VSAAHC-YKSRIQVRLGEHNIDVLEGNEQFINAAKIITHPNFNGNTLDNDIMLIKLSSPA

P00761.1 VSAAHC-YKSRIQVRLGEHNIDVLEGNEQFINAAKIITHPNFNGNTLDNDIMLIKLSSPA

Lunathrombase -----------------------------------IITHPNFNGNTLDNDIMLIK-----

********************

1H9H|E TLNSRVATVS-PRSCAAAGTECSLISGWGNTKSSGSSYPSLLQCSLKAPVLSDSSCKSSY

NP_001156363.1 TLNSRVATVSLPRSCAAAGTEC-LISGWGNTKSSGSSYPSLLQC-LKAPVLSDSSCKSSY

1EPT|B TLNSRVATVSLPRSCAAAGTEC-LISGWGNTK----------------------------

XP_020934119.1 TLNSRVATVSLPRSCAAAGTEC-LISGWGNTKSSGSSYPSLLQC-LKAPVLSDSSCKSSY

P00761.1 TLNSRVATVSLPRSCAAAGTEC-LISGWGNTKSSGSSYPSLLQC-LKAPVLSDSSCKSSY

Lunathrombase ------------------------------------------------------------

1H9H|E PGQITGNMICVGFLEGGKDSCSQGDSGGPVVCSNGQLQGIVSWGYGCSAQKNKPGVYTKV

NP_001156363.1 PGQITGNMICVGFLEGGKDSC-QGDSGGPVVC-NGQLQGIVSWGYGCA-QKNKPGVYTKV

1EPT|B ------------------------------------------------------------

XP_020934119.1 PGQITGNMICVGFLEGGKDSC-QGDSGGPVVC-NGQLQGIVSWGYGCA-QKNKPGVYTKV

P00761.1 PGQITGNMICVGFLEGGKDSC-QGDSGGPVVC-NGQLQGIVSWGYGCA-QKNKPGVYTKV

Lunathrombase ------------------------------------------------------------

1H9H|E CNYVNWIQQTIAAN

NP_001156363.1 CNYVNWIQQTIAAN

1EPT|B --------------

XP_020934119.1 CNYVNWIQQTIAAN

P00761.1 CNYVNWIQQTIAAN

Lunathrombase --------------

**Supplementary Fig. S2.** Time- dependent anticoagulant activity by lunathrombase (400 nM) against human PPP. Ca- Clotting time of PPP under identical experimental conditions (control) was found to be 96.8 ± 1.8 s. All values are means ± S.D. of three independent experiments. * P< 0.05.


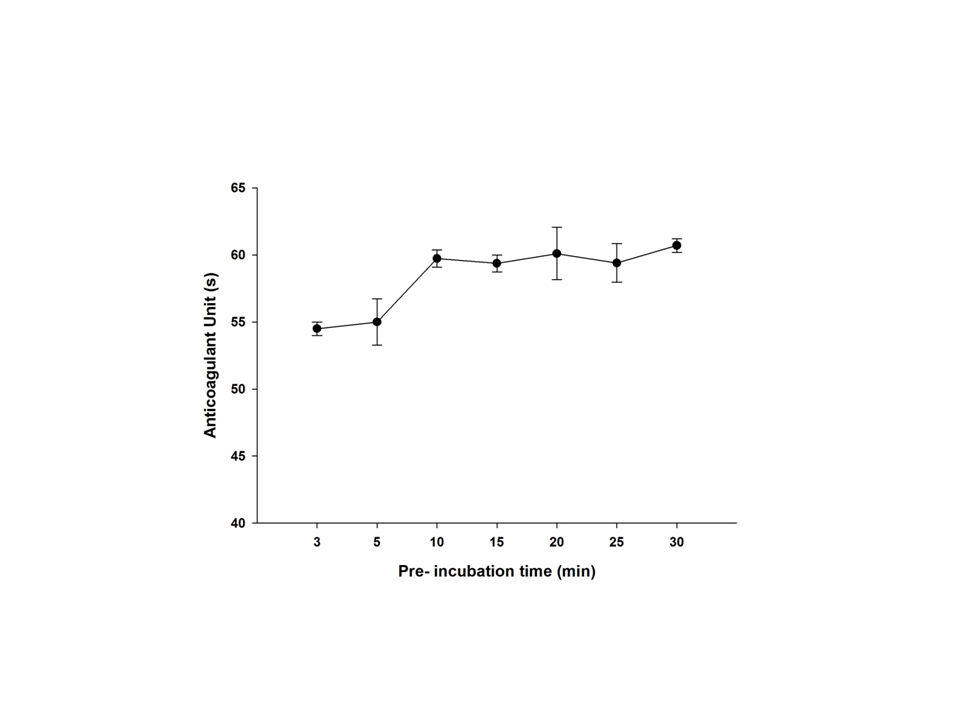


**Supplementary Fig. S3A.** A comparison of fibrinogenolytic activity among lunathrombase, Nattokinase, plasmin, and thrombin under identical conditions by 12.5% SDS-PAGE analysis. Lane 1, control human fibrinogen (0.25% w/v in 20 mM K-phosphate buffer, 150 mM NaCl, pH 7.4); lanes 2- 3, human fibrinogen treated with lunathrombase (0.2 µM); lanes 4- 5, human fibrinogen treated with Nattokinase (0.2 µM); lanes 6-7, human fibrinogen treated with plasmin (0.2 µM); lanes 8-9, human fibrinogen treated with thrombin (0.2 µM) for 15, 30 min, respectively, at 37 ºC, pH 7.4. **B.**  Densitometry analysis to determine the percent degradation of fibrinogen. All values are means ± S.D. of three independent experiments. Significance of difference with respect to Nattokinase, plasmin and thrombin, * p< 0.05.

A


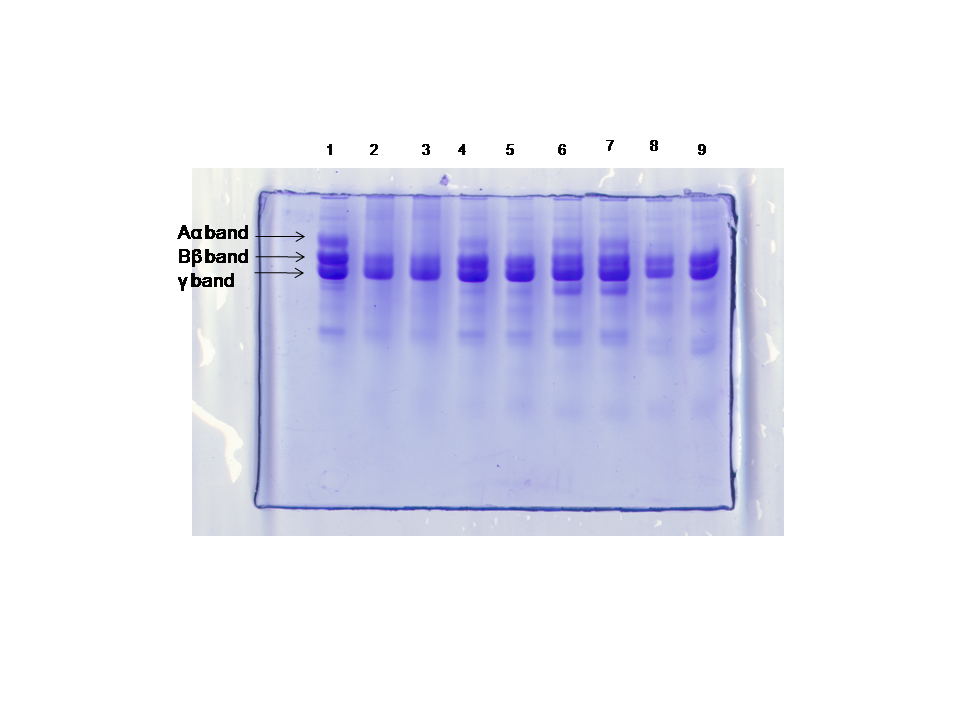


B


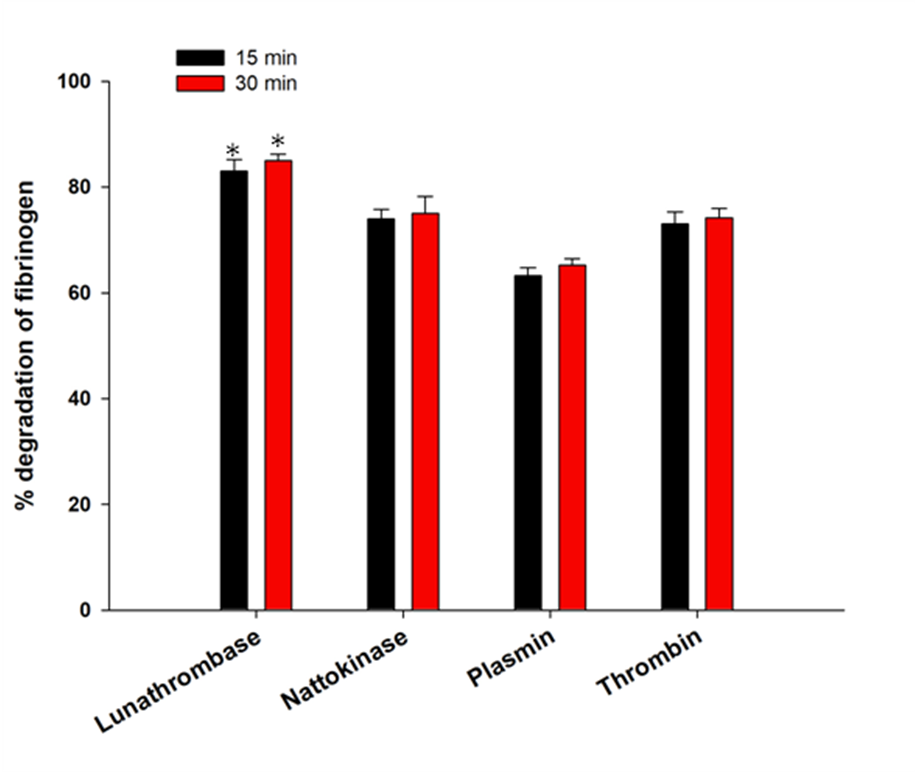


**Supplementary Fig. S4.** Determination of fibrinolytic activity of lunathrombase, Nattokinase, streptokinase, and plasmin under identical experimental conditions by 12.5% SDS-PAGE analysis. Lane 1, control human fibrin (0.25% w/v in 20 mM K-phosphate buffer, 150 mM NaCl, pH 7.4); lanes 2- 3, human fibrin degradation by lunathrombase (0.2 µM); lanes 4- 5, human fibrin degradation by Nattokinase (0.2 µM); lanes 6-7, human fibrin degradation by streptokinase (0.2 µM); lanes 8-9, human fibrin degradation by plasmin (0.2 µM) at 15, 30 min of incubation, respectively, at 37 ºC, pH 7.4. **B.**  Densitometry analysis to determine the percent degradation of fibrin. All values are means ± S.D. of three independent experiments. Significance of difference with respect to Nattokinase, streptokinase and plasmin, * p< 0.05.

A


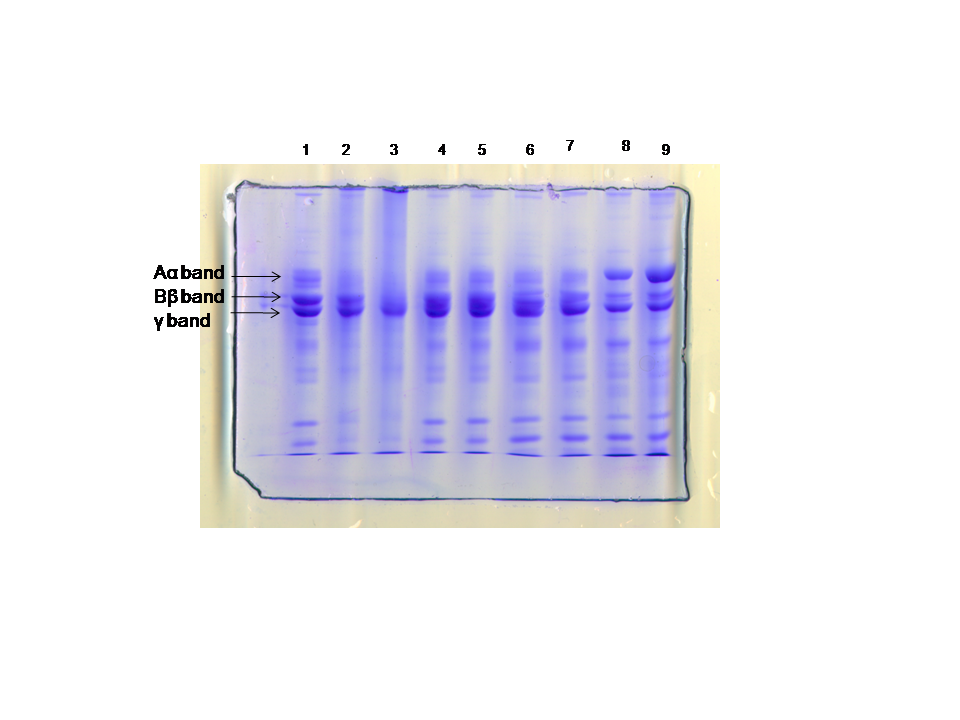


B


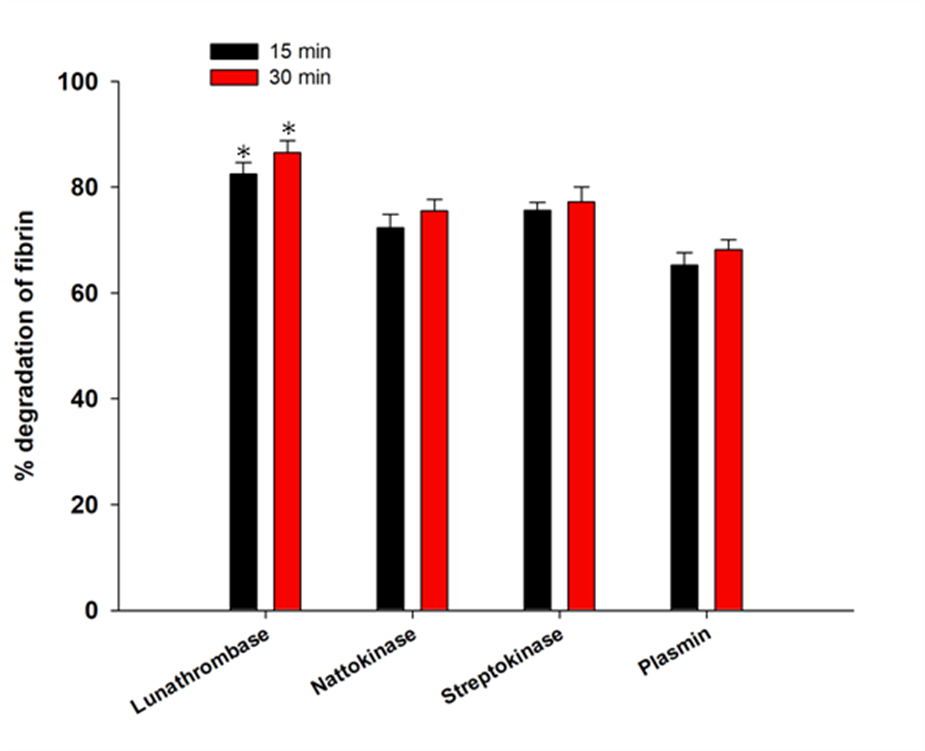


**Supplementary Fig. S5.** Determination of *Km* and *Vmax* values of lunathrombase against fibrinogen. Lineweaver-Burk plot and Michaelis-Menton plot (inset) of lunathrombase against fibrinogen.


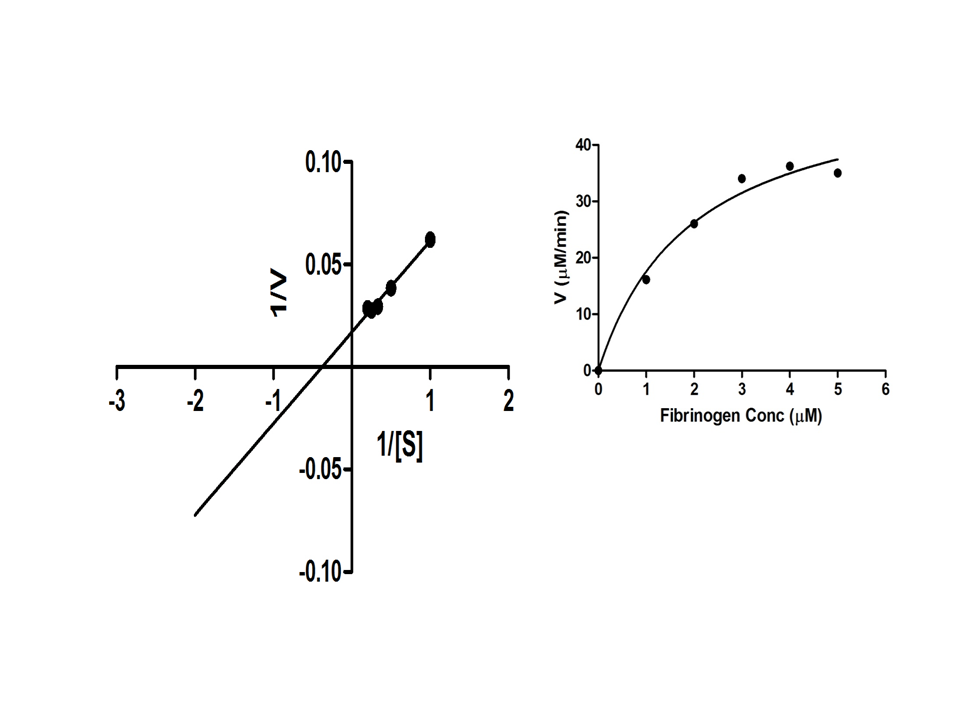


**Supplementary Fig. S6.** Determination of *Km* and *Vmax* values of Nattokinase against fibrinogen**.** Lineweaver-Burk plot and Michaelis-Menton plot (inset) of Nattokinase against fibrinogen


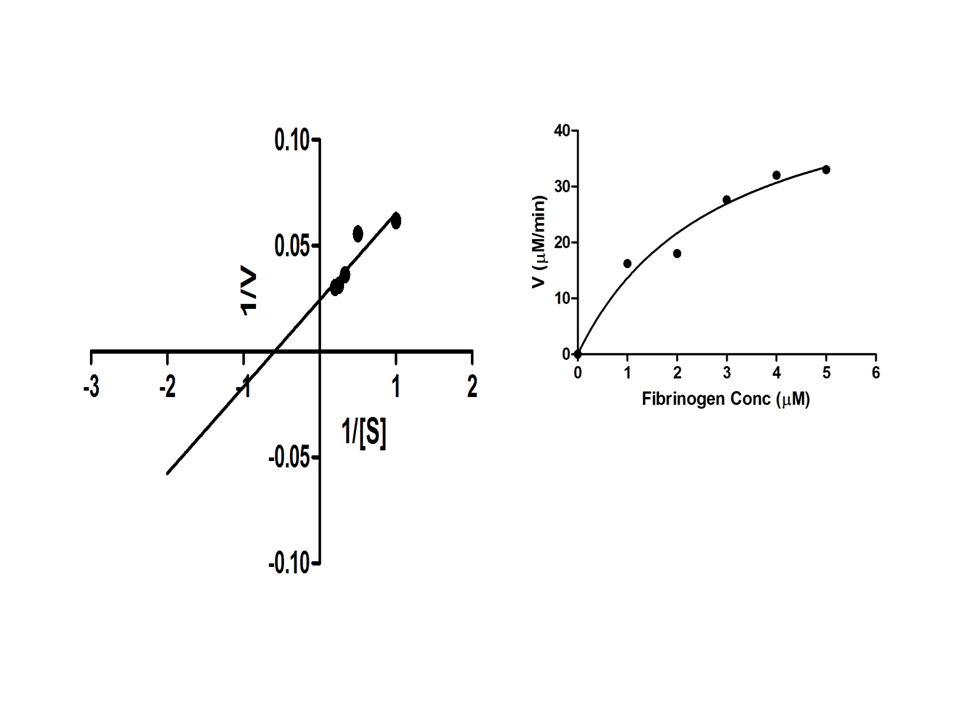


**Supplementary Fig. S7.** A comparison of fibrinogen degradation between Nattokinase and lunathrombase under identical experimental conditions by RP-UHPLC analysis. Human fibrinogen solution (2.5 mg/ml in 20 mM potassium phosphate buffer containing 100 mM NaCl, pH 7.4) was incubated with 0.2 µM of lunathrombase or Nattokinase for 15 min at 37 °C. The experiment was done as described in text.


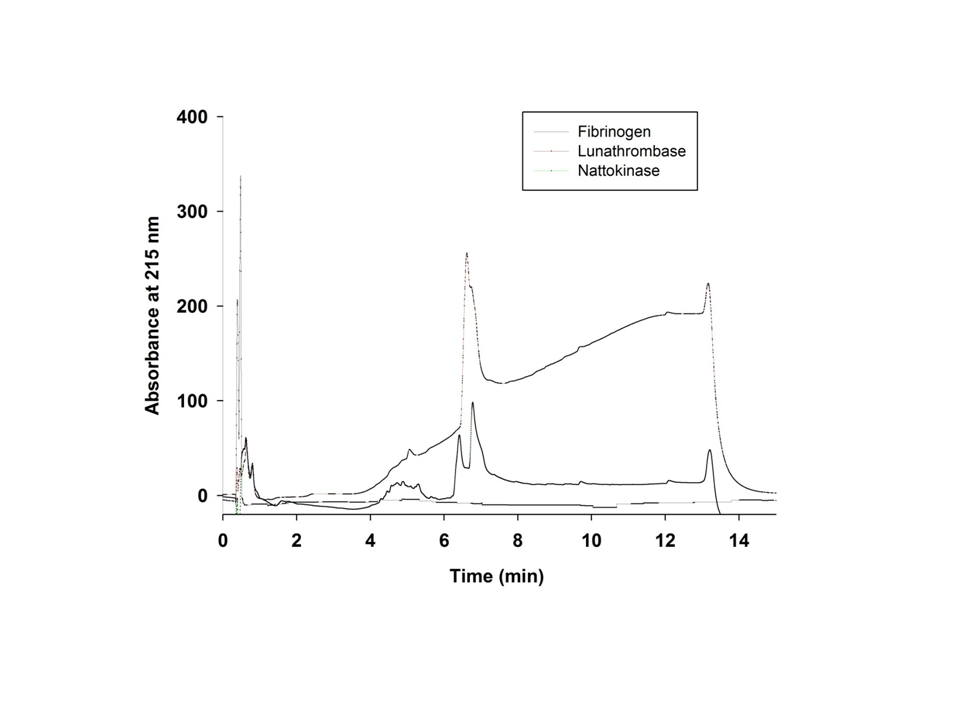


**Supplementary Fig. S8.** Effect of temperature (10-80 ºC) on fibrin(ogen)oytic activity of lunathrombase. The values are mean ± S.D. of triplicate determinations.


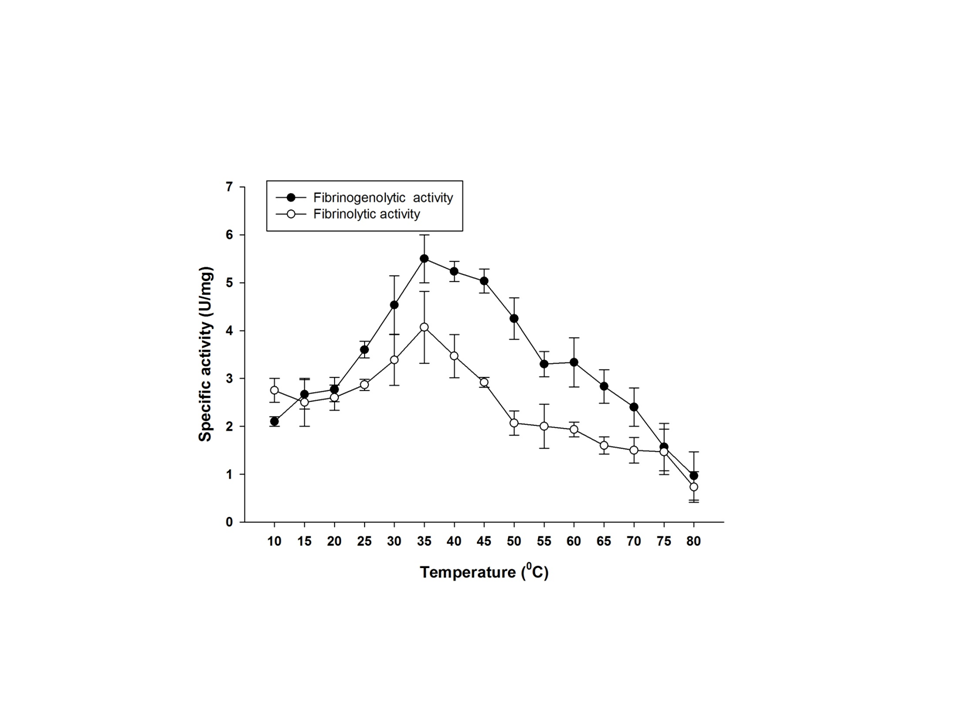


**Supplementary Fig. S9.** Influence of pH (2-12) on fibrin(ogen)oytic activity of lunathrombase. The values are mean ± S.D. of triplicate determinations.


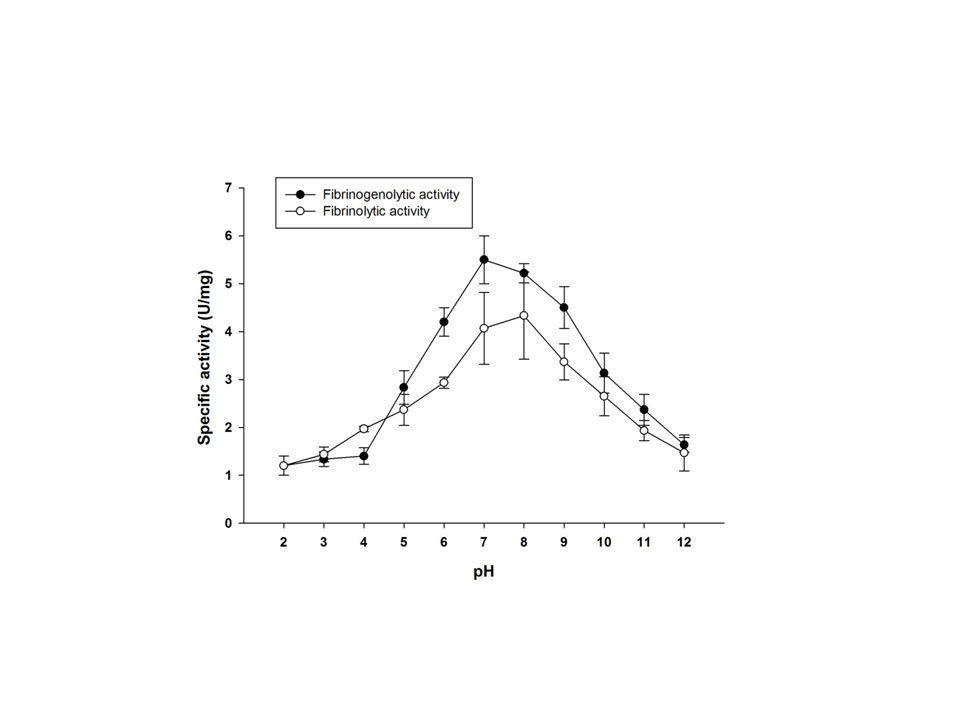


**Supplementary Fig. S10.** Determination of glycosylation in lunathrombase. Lane 1, protein marker; lane 2, lunathrombase (20 µg, denatured) (lunathrombase heated at 100 °C for 10 min) and incubated at 37 °C for 4 hour; Lane 3 & 4, denatured lunathrombase (20 µg) treated with PNGase (500 units) and neuraminidase, respectively, for 4 hour at 37 °C; Lane 5, native lunathrombase (20 µg).


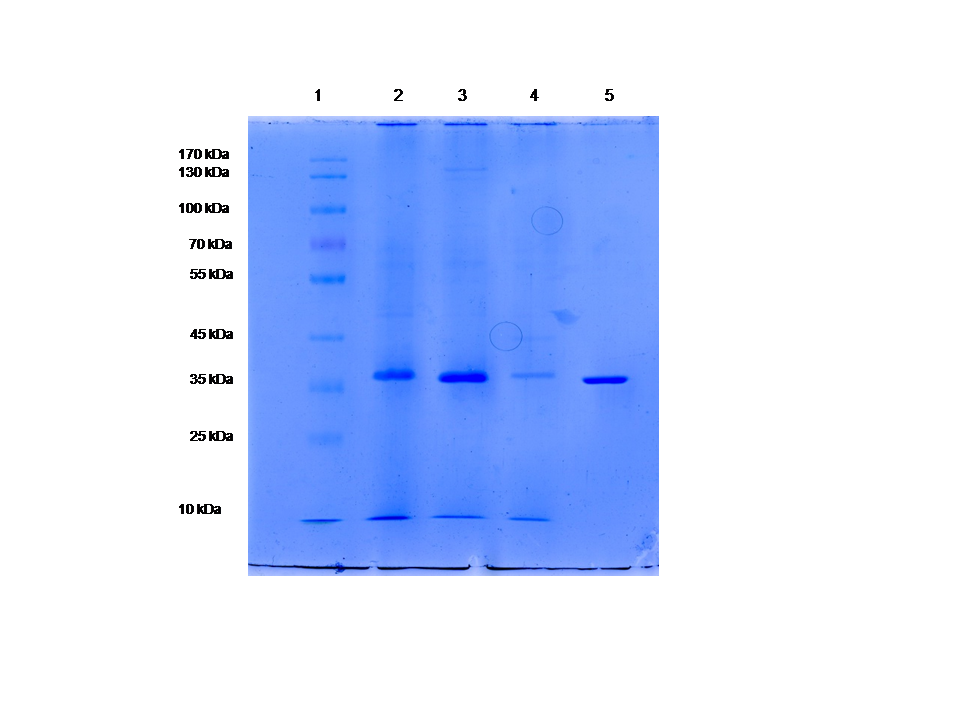


**Supplementary Fig. S11.** Effect of metal ions (2 mM) on fibrin(ogen)oytic activity of lunathrombase. The values are mean ± S.D. of triplicate determinations.


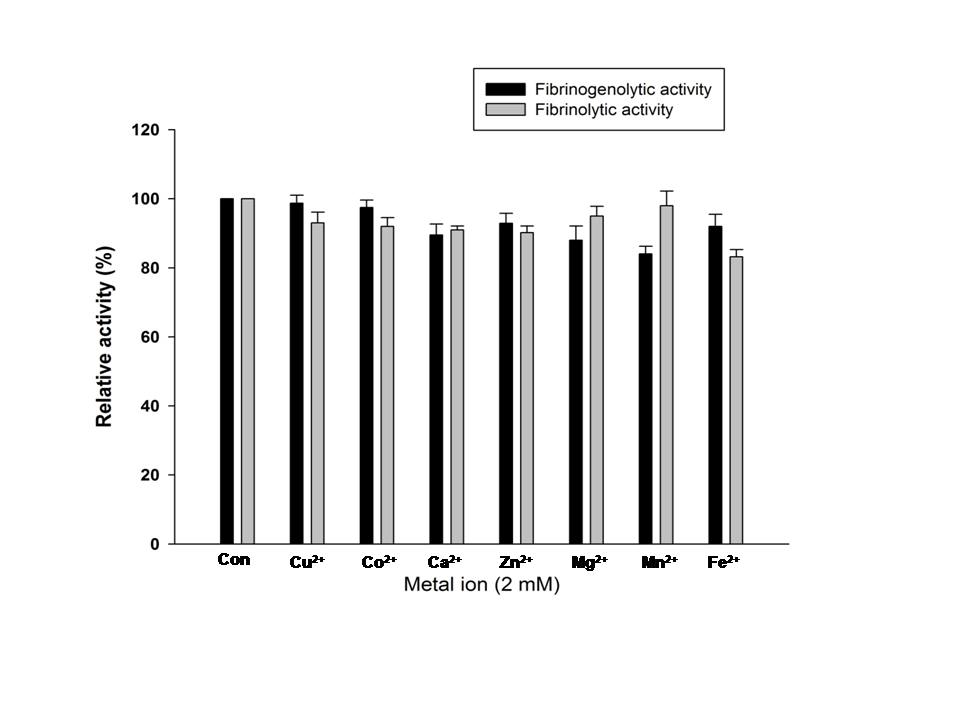


**Supplementary Fig. S12.** The effect of inhibitors on fibrinogenolytic activity of lunathrombase. The degradation products were separated by 12.5% SDS-PAGE (reducing conditions). Lane 1, control human fibrinogen (0.25% w/v in 20 mM K-phosphate buffer, 150 mM NaCl, pH 7.4); lanes 2-8, human fibrinogen degradation by lunathrombase (0.2 µM) pre-incubated with 4 mM DTT, EDTA, TPCK, TLCK, PMSF, IAA and pBPB, respectively, for 60 min at 37 ºC, pH 7.4.


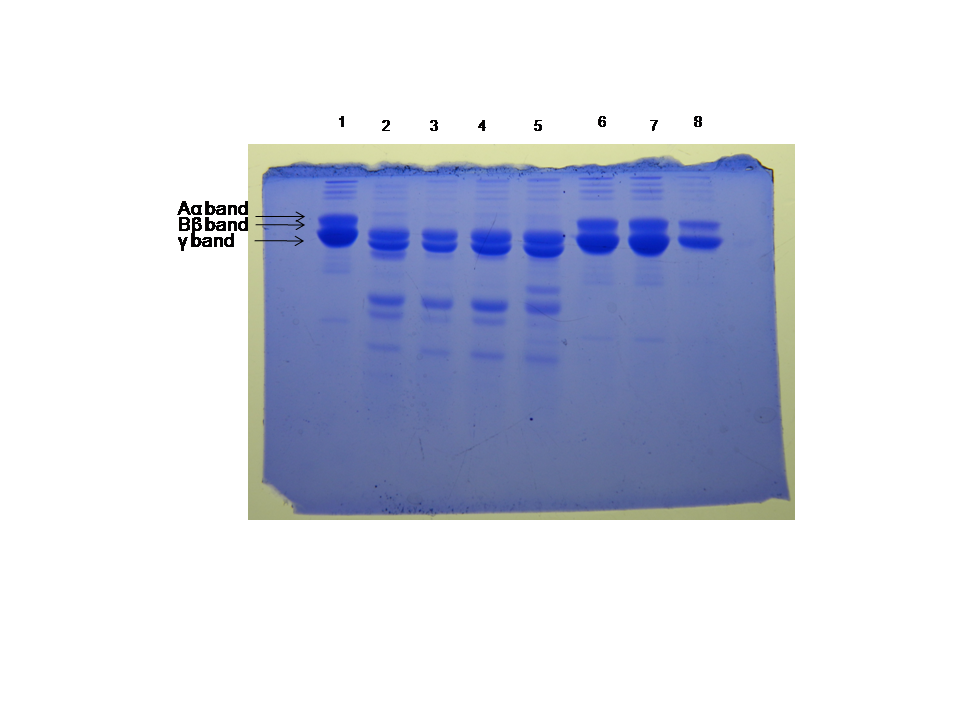


**Supplementary Fig. S13**. Degradation of flbronectin, laminin and type-IV collagen by lunathrombase. ECM proteins (laminin, type-IV colIagen, and fibronectin) were incubated with lunathrombase in a substrate: enzyme ratio of 15: I (w/w) at 37 °C, pH 7.4 for 12 h and the degradation products were analyzed by 10% SDS- PAGE (reduced conditions). Lane 1 and 6, protein molecular markers; lanes 2, 4, and 7 control laminin, type-IV collagen, and fibrinonectin respectively; lanes 3, 5, and 8 laminin, type-IV collagen, and fibronectin, respectively treated with lunathrombase (0.2 µM).


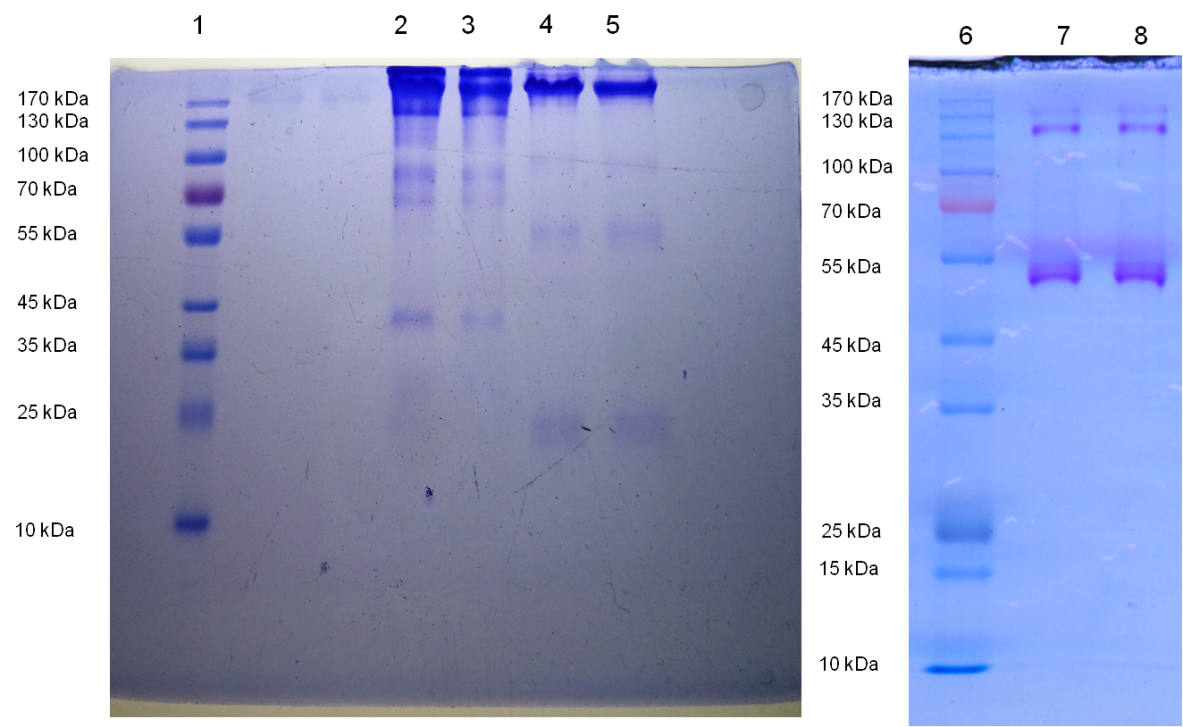


**Supplementary Fig. S14**. A comparison of *in vitro* thrombolytic activity of lunathrombase (1 µM), streptokinase (1 µM), plasmin (1 µM), Nattokinase (1 µM) and tissue plasminogen activator (tPA) (1 µM) under identical experimental conditions on heated and non-heated blood clots. Values are means ± S.D. of triplicate determinations. Significance of difference with between heated and non heated blood clot dissolving property by lunathrombase, *p< 0.05


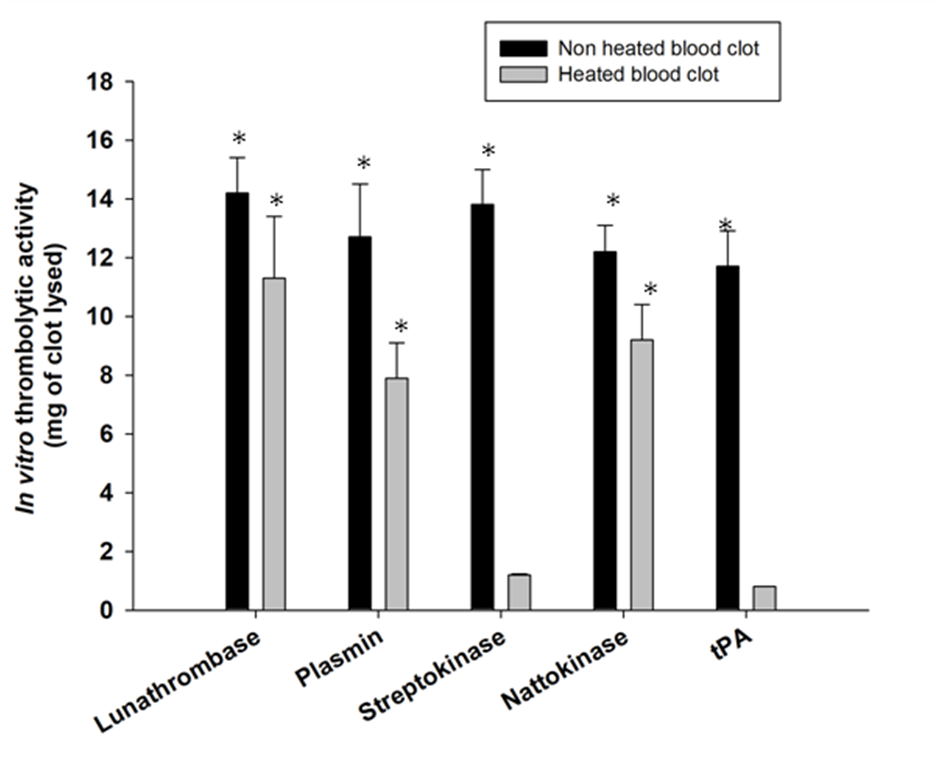


**Supplementary Fig. S15.** *In vitro* cell viability by MTT assay. HEK 293 cells were treated with lunathrombase (0- 2.0 µM) for 24 h at 37 ˚C. All values are mean ± SD of triplicate determinations.


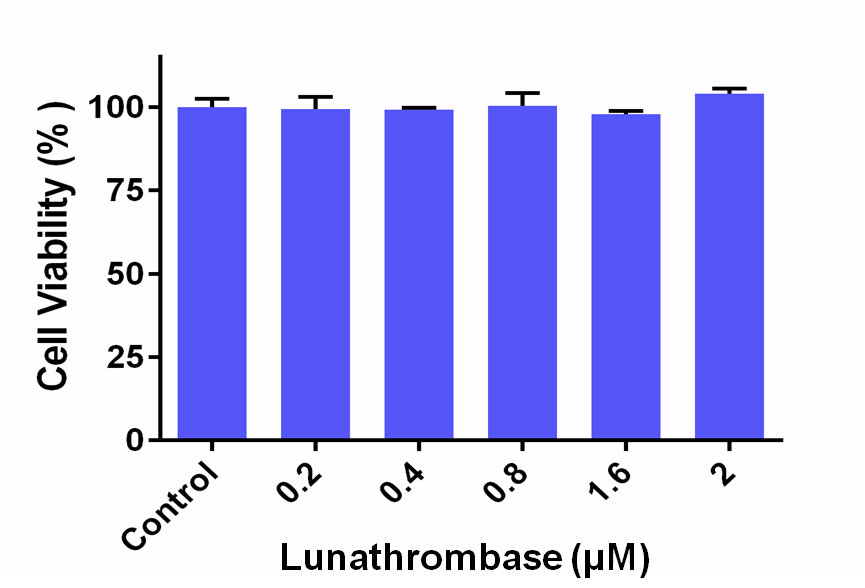


**Supplementary Fig. S16.** Calcein-AM staining of HEK 293 cells. **A.** HEK 293 cells treated with control media. **B.** HEK 293 cells treated with 1X PBS, pH 7.4 (without lunathrombase), and **C.** HEK 293 cells treated with lunathrombase (2.0 µM) for 24 h at 37 ºC and then stained with Calcein-AM for 5 min. Green fluoresces indicating live cells.

**
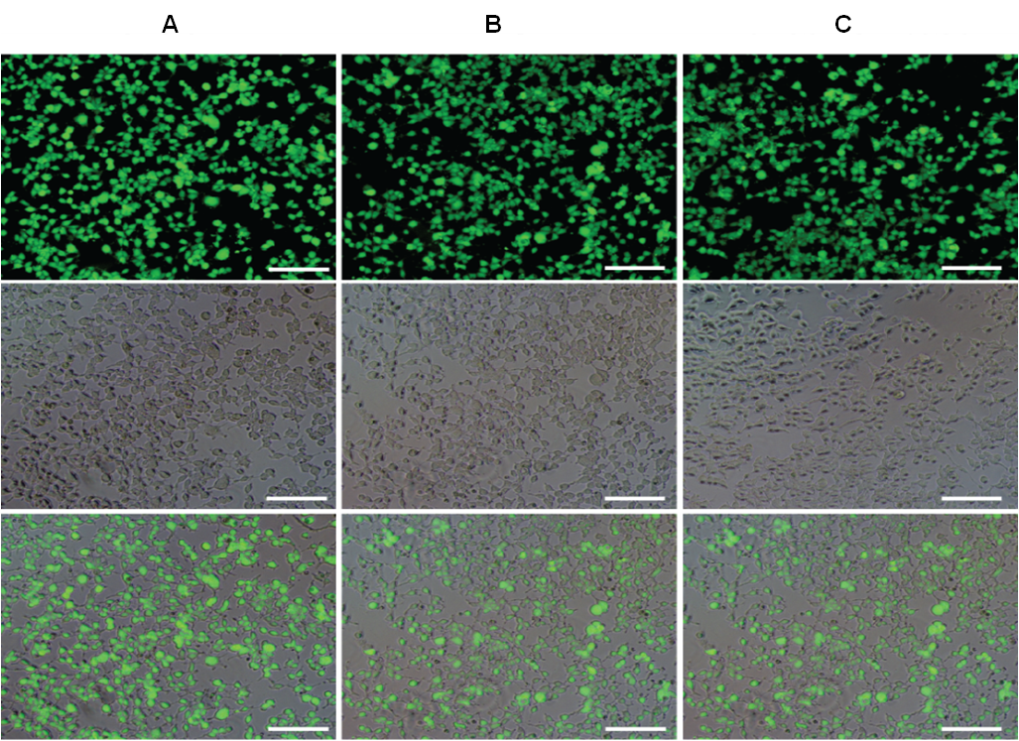
**

**Supplementary Fig. S17**. Cell cycle analysis using propidium iodide (PI) staining and flow cytometry. HEK 293 cells (1.5 x 10^5^ cells per ml) were treated for 24 h at 37 ˚C with or without lunathrombase (2.0 µM). Cells were harvested using trypsinization and stained with PI for 2 h and analyzed by flow cytometry.

**
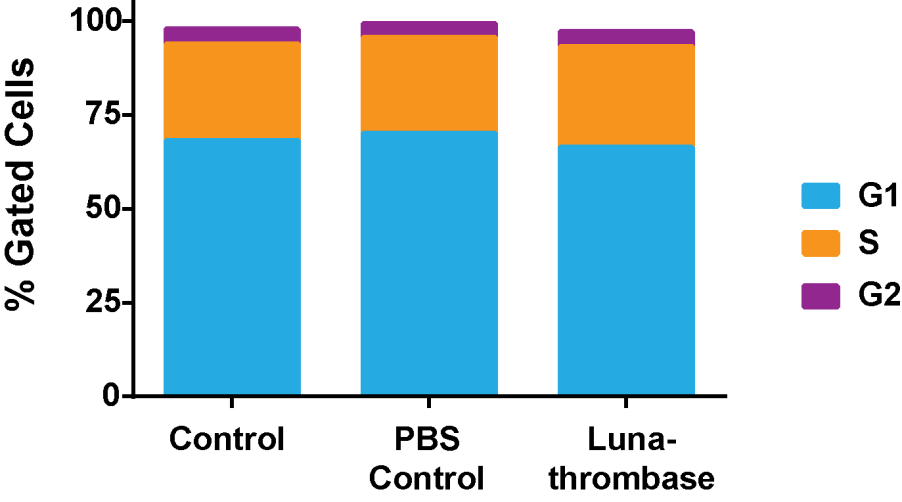
**

**Supplementary Fig. S18:** A comparison of properties between native and catalytically inactive lunathrombase. **A.** platelet deaggregation activity~~.~~ **B**. inhibition of collagen-induced platelet aggregation, **C.** inhibition of ADP-induced platelet aggregation, and **D.** inhibition of arachidonic acid-induced platelet aggregation. Values are mean ± SD of triplicate determinations. Significance of difference with respect to native lunathrombase. * p <0.05


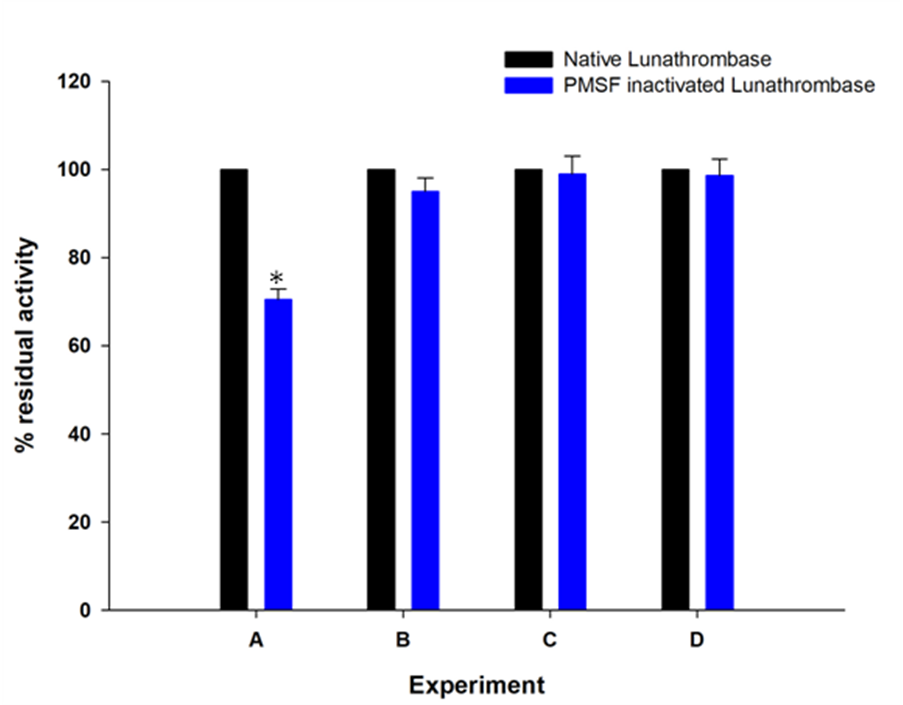


**Supplementary Fig. 19:** Percent aggregation / deaggregation of chymotrypsin-treated or untreated washed platelets (1X10^6^ cells). Effect of **(A)** fibrinogen (0.2 µM), **(C)** lunathrombase (0.2 µM), and **(E)** catalytically inactive lunathrombase (0.2 µM) on washed untreated (control) platelets. Effect of **(B)** fibrinogen (0.2 µM), **(D)** lunathrombase (0.2 µM), **(F)** catalytically inactive lunathrombase (0.2 µM) and **(G)** BSA (0.2 µM) on washed platelets pre-treated with α-chymotrypsin for 15 min. Values are mean ± SD of three determinations.


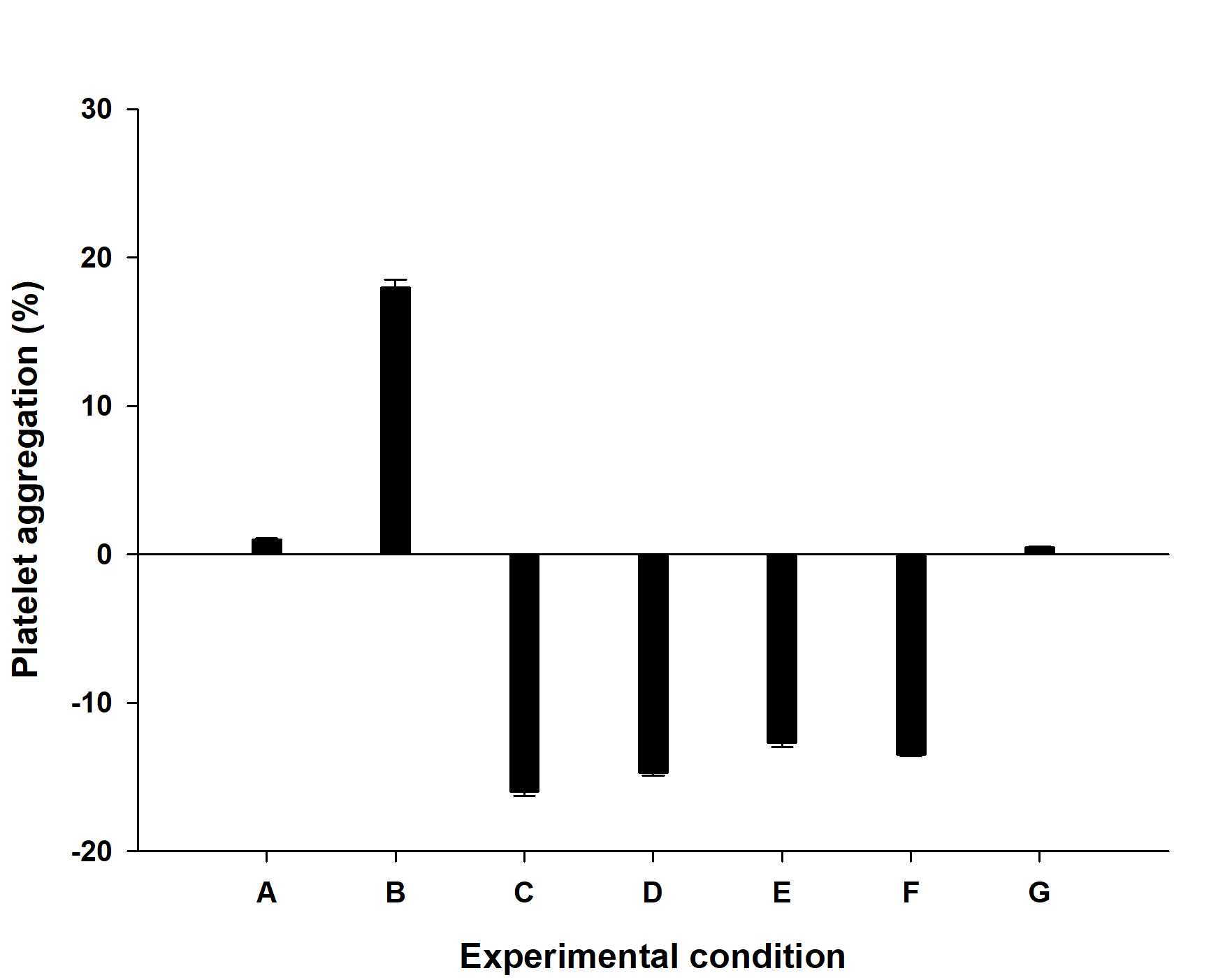


**Supplementary Fig. S20.** Binding of PMSF-inactivated lunathrombase (0.2 -1.0 µM) to **A.** human fibrinogen (1000 ng), and **B.** human platelet GPIIb/IIIa receptor. Values are mean ± SD of triplicate determinations.

A


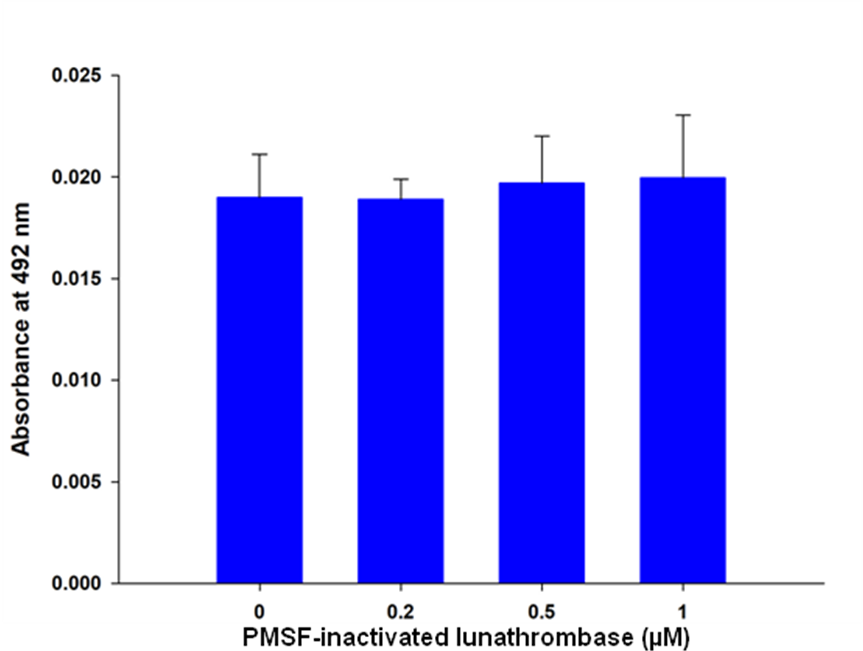


B


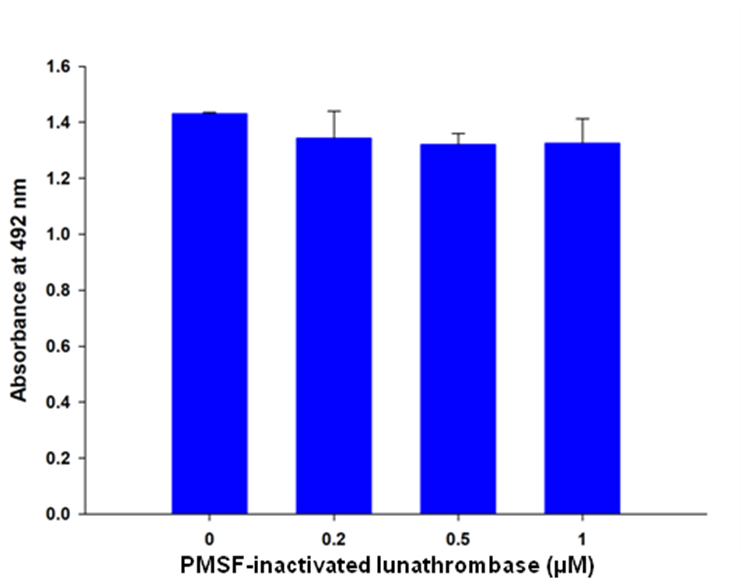


**Supplementary Fig. S21.** Determination of *Km* and *Vmax* values of lunathrombase against COX-1 enzyme**.** Lineweaver-Burk plot and Michaelis-Menton plot (inset) of lunathrombase against COX-1.


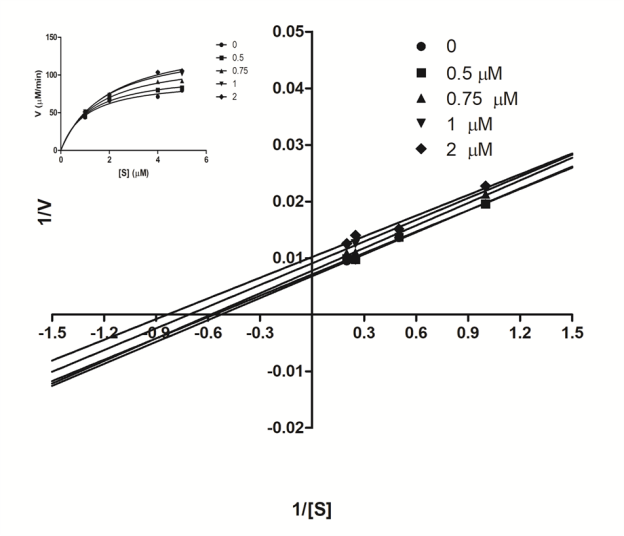


**Supplementary Fig. S22**. The original gel of figure 1d. Determination of purity and molecular mass of AF_GF3 (lunathrombase) by 12.5% SDS-PAGE; Lane 1, protein molecular markers; lane 2, reduced lunathrombase (20.0 µg).

**
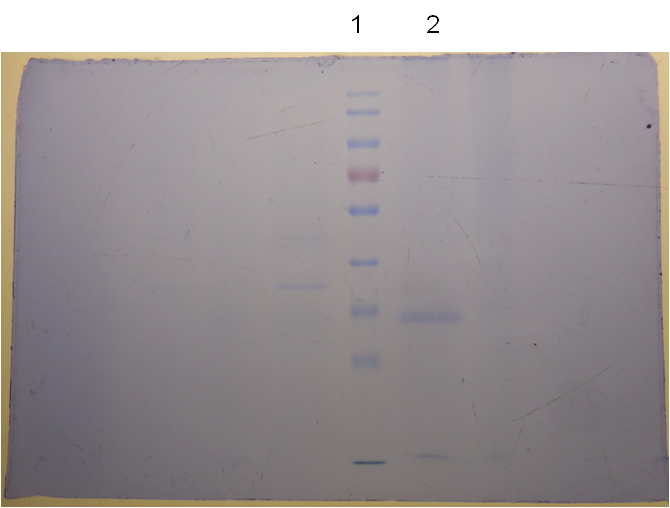
**

**Supplementary Table S1. A summary of purification of lunathrombase from aqueous leaves extract of *L. indica*. Values are mean ± S.D. of triplicate determinations. Significance of difference with respect to crude aqueous extract, * *p* < 0.01**

| **Fraction**  **(1.0 µg/ml)** | **Specific activity (Units/mg)** | |
| --- | --- | --- |
|  | **Anticoagulant^a^** | **Fibrinogenolytic^b^** |
| Crude aqueous leave extract | 30.5 x 10^3^ ± 1.00 | 8.10 ± 0.64 |
| Anion exchange unbound fraction (AEX_1) | 34.06 x 10^3^ ± 1.00 * | 7.90 ± 0.30 |
| Cation exchange unbound fraction (CEX_1) | 46.4 x 10^4^ ± 2.8 * | 12.13 ± 1.02 * |
| Gel filtrations |  |  |
| AF_GF1 | 15.5 x 10^3^ ± 2.5 | 4.20 ± 1.6 |
| AF_GF2 | 12.3 x 10^3^ ± 1.5 | 2.20 ± 1.3 |
| AF_GF3 (lunathrombase) | 32.5 x 10^3^ ± 3.5 | 5.50 ± 1.4 |

**Supplementary Table S2.** Amino acids composition of lunathrombase (% nmol). Values are mean of triplicate determinations.

| **Amino acids** | **% nM** |
| --- | --- |
| ASP | 4.2 |
| HIS | 5.0 |
| GLU | 23.1 |
| SER | 1.1 |
| CYS | 1.5 |
| ARG | 1.0 |
| TYR | 2.6 |
| VAL | 22.0 |
| MET | 3.0 |
| PHE | 2.0 |
| ILE | 1.3 |
| LEU | 1.8 |
| LYS | 30.0 |
| PRO | 1.4 |

**Supplementary Table S3.** Effect of chemical inhibitors on fibrinogenolytic activity of lunathrombase. Values represent mean ± SD of three determinations. Significance of difference with respect to control. * p < 0.01, ** p < 0.05

| **Inhibitors** | **Relative activity (%)** |
| --- | --- |
| Control (without inhibitor) | 100 |
| Inhibitors (concentrations) | |
| PMSF (2 mM) | 23.69 ± 1.3 ** |
| PMSF (4 mM) | 18.81 ± 2.2 * |
| IAA (2 mM) | 48.15 ± 2.7 ** |
| IAA (4 mM) | 38.40 ± 3.1 * |
| pBPB (2 mM) | 53.38 ± 2.1 ** |
| pBPB (4 mM) | 43.12 ± 1.7** |
| TPCK (100 µM) | 87.20 ± 1.1 |
| TLCK (100 µM) | 85.23 ± 2.3 |
| DTT (4 mM) | 91.51 ± 3.5 |
| EDTA (4 mM) | 98.24 ± 5.1 |

**Supplementary Table S4**. Serum hematological parameters of lunathrombase-treated (10 mg/kg) and control group of rats after 72 hours of *i.p.* injection. Values are mean ± SD of 6 rats. There was no significant difference of values (p>0.05) between control, and lunathrombase-treated groups of rats.

|  | **Values** | |
| --- | --- | --- |
| **Parameters (Unit)** | **Control** | **lunathrombase-treated (10 mg/kg)** |
| WBC(m/mm^3^) | 5.06 ± 0.36 | 4.85 ± 0.98 |
| Lymphocytes(%) | 36.17 ± 3.00 | 30.60 ± 0.61 |
| Monocytes(%) | 5.67 ± 0.20 | 5.13 ± 0.06 |
| Neutrophils(%) | 50.63 ± 6.62 | 61.33 ± 0.31 |
| Eosinophils(%) | 10.63 ± 0.26 | 12.20 ± 0.10 |
| Basophils(%) | 0.10 ± 0.00 | 0.17 ± 0.12 |
| Total RBC(m/mm^3^) | 8.77 ± 0.12 | 7.65 ± 0.53 |
| MCV(fl) | 44.23 ± 2.60 | 48.77 ± 1.99 |
| HCt(%) | 36.20 ± 0.40 | 35.17 ± 1.77 |
| MCH(pg) | 15.77 ± 0.63 | 18.60 ± 2.12 |
| MCHC(g/dl) | 37.13 ± 0.66 | 31.17 ± 4.80 |
| RDW | 15.73 ± 0.16 | 12.70 ± 2.00 |
| Hb(g/dl) | 12.73 ± 0.25 | 12.67 ± 0.64 |
| MPV(fl) | 6.93 ± 0.10 | 6.80 ± 0.17 |
| PCt(%) | 0.31 ± 0.11 | 0.29 ± 0.03 |
| PDW | 8.37 ± 0.16 | 8.07 ± 0.25 |

**Supplementary Table S5**. Some biochemical properties of serum of control and lunathrombase-treated (10 mg/kg) rats after 72 hours of *i.p*. injection. Values are mean ± SD of 6 rats. There was no significant difference of values (p>0.05) between control and lunathrombase-treated groups of rats.

|  | **Values** | |
| --- | --- | --- |
| **Parameters (Unit)** | **Control** | **lunathrombase-treated** |
| LDL(mg/dl) | 13.30 ± 0.60 | 13.20 ± 0.50 |
| HDL(mg/dl) | 18.80 ± 0.40 | 16.60 ± 1.10 |
| Glucose(mg/dl) | 70.00 ± 5.20 | 67.00 ± 5.50 |
| Urea(mg/dl) | 53.80 ± 3.20 | 53.00 ± 3.30 |
| Total protein(g/dl) | 6.30 ± 0.40 | 6.10 ± 0.30 |
| Uric acid(mg/dl) | 1.30 ± 0.40 | 1.30 ± 0.20 |
| Triglycerides | 52.10 ± 2.60 | 50.40 ± 3.20 |
| Cholesterol (mg/dl) | 46.80 ± 3.40 | 48.20 ± 2.10 |
| Creatinine(mg/dl) | 1.00 ± 0.20 | 1.40 ± 0.20 |
| SGPT(U/L) | 91.30 ± 6.60 | 95.40 ± 1.70 |
| SGOT(U/L) | 162.30 ± 4.90 | 166.50 ± 3.10 |
